# Spider crabs of the Western Atlantic with special reference to fossil and some modern Mithracidae

**DOI:** 10.7717/peerj.1301

**Published:** 2015-10-01

**Authors:** Adiël A. Klompmaker, Roger W. Portell, Aaron T. Klier, Vanessa Prueter, Alyssa L. Tucker

**Affiliations:** 1 Florida Museum of Natural History, University of Florida, Gainesville, FL, United States of America; 2 Department of Geological Sciences, University of Florida, Gainesville, FL, United States of America; 3 Department of Biology, University of Florida, Gainesville, FL, United States of America

**Keywords:** Biodiversity, Paleontology, Decapoda, Crustacea, Biogeography, Taxonomy, Majoidea, Systematics, Paleoecology, Reef

## Abstract

Spider crabs (Majoidea) are well-known from modern oceans and are also common in the western part of the Atlantic Ocean. When spider crabs appeared in the Western Atlantic in deep time, and when they became diverse, hinges on their fossil record. By reviewing their fossil record, we show that (1) spider crabs first appeared in the Western Atlantic in the Late Cretaceous, (2) they became common since the Miocene, and (3) most species and genera are found in the Caribbean region from the Miocene onwards. Furthermore, taxonomic work on some modern and fossil Mithracidae, a family that might have originated in the Western Atlantic, was conducted. Specifically, *Maguimithrax* gen. nov. is erected to accommodate the extant species *Damithrax spinosissimus*, while *Damithrax* cf. *pleuracanthus* is recognized for the first time from the fossil record (late Pliocene–early Pleistocene, Florida, USA). Furthermore, two new species are described from the lower Miocene coral-associated limestones of Jamaica (*Mithrax arawakum* sp. nov. and *Nemausa windsorae* sp. nov.). Spurred by a recent revision of the subfamily, two known species from the same deposits are refigured and transferred to new genera: *Mithrax donovani* to *Nemausa*, and *Mithrax unguis* to *Damithrax*. The diverse assemblage of decapods from these coral-associated limestones underlines the importance of reefs for the abundance and diversity of decapods in deep time. Finally, we quantitatively show that these crabs possess allometric growth in that length/width ratios drop as specimens grow, a factor that is not always taken into account while describing and comparing among taxa.

## Introduction

Modern spider crabs (Majoidea) range in size from a few millimeters to more than a meter in carapace length (Griffin, 1966). Long, slender legs and a pyriform to triangular shape give many of them a spider-like appearance. They occur in nearly all oceans (e.g., Griffin, 1966), and many of them have been found to decorate themselves for camouflage (e.g., Wicksten, 1993; Guinot, Tavares & Castro, 2013). Today, spider crabs are very diverse with nearly 1,000 species worldwide (Ng, Guinot & Davie, 2008; De Grave et al., 2009). More than 125 species have been found in the fossil record (De Grave et al., 2009; Schweitzer et al., 2010), with the oldest species known from the mid-Cretaceous of Europe (Breton, 2009; Klompmaker, 2013). Collins, Portell & Donovan (2009) provided an overview of fossil decapods, including majoids, known from the Caribbean region. Since then, various new fossil majoid occurrences and new fossil species have been reported for the Western Atlantic (e.g., Collins et al., 2010; Collins & Donovan, 2012; Feldmann et al., 2013; Franţescu, 2013; Varela, 2013; Collins, Garvie & Mellish, 2014; Stepp, 2014).

The Mithracidae sensu Windsor, 2010, and Windsor & Felder, 2014 (=Mithracinae sensu Ng, Guinot & Davie, 2008, and De Grave et al., 2009) are found in (sub)tropical waters from intertidal to 450 m depth, mainly as reef- and rubble dwellers (Windsor & Felder, 2014). Recently, the family was revised extensively using morphological and molecular analyses resulting in numerous redefinitions and the resurrection and erection of four genera (Windsor & Felder, 2014). As is the case for the Western and Eastern Pacific, the Mithracidae are well-known from the Western Atlantic with over 30 species (e.g., Rathbun, 1925; Abele & Kim, 1986; Felder et al., 2009; Alves et al., 2012; Windsor & Felder, 2014), the latter authors (p. 154) suggesting it is an “amphi-American” group. Although their fossil record is decent with 25 known species (Schweitzer et al., 2010; Tables 1 and S1), mostly originating from the Western Atlantic (Table 1), additional research is required because representatives of many extant genera have a scarce fossil record.

**Table 1:** The number of reported fossil carapace specimens of species of Mithracidae worldwide and their country of origin and age. Note that Windsor & Felder (2014) ascribed *Stenocionops* Desmarest, 1823, and *Micippa* Leach, 1817, to Mithracidae s.l.; the fossil *Antarctomithrax* Feldmann, 1994, was not discussed.

Taxon	Number of fossil carapace specimens	Country of origin and age	References other than original description
*Antarctomithrax thomsoni Feldmann, 1994	1	Antarctica [Eocene]	
*Damithrax* cf. *pleuracanthus* (Stimpson, 1871)	1	USA (Florida) [late Pliocene—early Pleistocene]	herein
*Damithrax unguis (Portell & Collins, 2004)	24	Jamaica [early Miocene]	herein
*Maguimithrax spinosissimus* (Lamarck, 1818)	1	Barbados [Pliocene, Pleistocene], Curaçao [Pliocene], Jamaica [late Pleistocene]	Collins & Morris (1976); Morris (1993); Stepp (2014)
*Micippa annamariae Gatt & De Angeli, 2010	3	Malta [late Miocene]	
*Micippa antiqua Beschin, De Angeli & Checchi, 2001	2	Italy [early Oligocene]	De Angeli & Beschin (2008)
*Micippa hungarica (Lörenthey in Lörenthey & Beurlen, 1929) [=*Maia austriaca* Bachmayer, 1953; *Phrynolambrus weinfurteri* Bachmayer, 1953]	70	Austria [late Miocene], Hungary [middle Miocene], Poland [middle Miocene]	Bachmayer (1953); Müller (1974); Müller (1984); Müller (1996)
*Micippa* cf. *thalia* (Herbst, 1782–1804)	0 (rostral spines)	Japan [Pleistocene]	Kato & Karasawa (1998)
*Mithraculus* aff. *coryphe* (Herbst, 1782–1804)	1	Jamaica [late Miocene]	Collins et al. (2010)
*Mithraculus* cf. *forceps* Milne-Edwards, 1873–1880	5	Jamaica [late Pleistocene]	Morris (1993); Collins, Donovan & Stemann (2009)
*Mithrax aculeatus* (Herbst, 1782–1804) (=*Mithrax verrucosus* Milne-Edwards, 1832)	5	Barbados [Pliocene, Pleistocene], Jamaica [late Pleistocene]	Collins & Morris (1976); Morris (1993); Collins, Donovan & Stemann (2009)
*Mithrax arawakum sp. nov.	2	Jamaica [early Miocene]	herein
*Mithrax hemphilli* Rathbun, 1892	6	Barbados [Pleistocene]	Collins & Morris (1976)
*Mithrax “hispidus”* (Herbst, 1782–1804) [=*M. caribbaeus* Rathbun, 1920]	7	Barbados [Pliocene, Pleistocene], Cuba [Plio-Pleistocene], Jamaica [late Pleistocene]	Collins & Morris (1976); Morris (1993); Collins, Donovan & Dixon (1996); Varela & Rojas-Consuegra (2009); Varela & Rojas-Consuegra (2011)
*Nemausa acuticornis* (Stimpson, 1871)	2	Jamaica [late Pleistocene]	Collins, Donovan & Stemann (2009)
*Nemausa donovani (Portell & Collins, 2004)	1	Jamaica [early Miocene]	herein
*Nemausa windsorae sp. nov.	1	Jamaica [early Miocene]	herein
*Stenocionops coelatus* (Milne-Edwards, 1878)	0 (dactylus and propodus)	USA (North Carolina) [early Pliocene]	Young (2014)
*Stenocionops dyeri Blow, 2003	7	USA (Virginia) [Pliocene]	herein
*Stenocionops primus Rathbun, 1935	0 (propodus)	USA (Arkansas) [Santonian]	
*Stenocionops suwanneeana Rathbun, 1935	0 (propodus)	USA (Florida) [late Eocene]	Portell (2004)
*Teleophrys acornis Portell & Collins, 2004	1	Jamaica [early Miocene]	
*Teleophrys ruber* (Stimpson, 1871)	1	Barbados [Pliocene]	Collins & Morris (1976)
*Thoe asperoides Collins & Todd (in Todd & Collins, 2005)	1	Panama [late Miocene], Costa Rica [late Pliocene—early Pleistocene]	
*Thoe vanuaensis (Rathbun, 1945)	0 (left chela)	Fiji [Pliocene]	

**Notes.**

* Denotes exclusively fossil mithracid species.

Their limited fossil record is expressed clearly in the low number of specimens available for fossil Mithracidae. Table 1 shows that, on average, only about six carapaces are available per species for those that have a fossil record, with the average being highly skewed by *Damithrax unguis* (Portell & Collins, 2004) and *Micippa hungarica* (Lörenthey in Lörenthey & Beurlen, 1929). In fact, 18/25 species are represented by no more than three carapaces. This severely hampers our understanding of intraspecific variation, possible sexual dimorphism, and ontogenetic variation, all of which are important factors when defining a species. As quantitatively shown, carapace length/width ratios are known to change throughout ontogeny for a variety of fossil crabs (e.g., Klompmaker, Feldmann & Schweitzer, 2012; De Jesús Gómez-Cruz, Bermúdez & Vega, 2015), and allometric growth was recently found for fossil ghost shrimp claws as well (e.g., Klompmaker et al., 2015). Although recent diagnoses of fossil crab genera and species usually contain statements about length/width ratios (e.g., Fraaije et al., 2010; Feldmann et al., 2011; Hyžný & Schlögl, 2011; Klompmaker, Artal & Gulisano, 2011; Klompmaker et al., 2011; Schweitzer & Feldmann, 2011; Luque et al., 2012; Starzyk, Krzemińska & Krzemiński, 2012; Vega et al., 2012; Artal, Van Bakel & Onetti, 2014; Ossó & Díaz Isa, 2014; Kočová Veselská, Kočí & Kubajko, 2014; Beschin, Busulini & Tessier, 2015), possible changes to this ratio as the animal grows were not always studied, in part due to a limited number of specimens. Possible allometric growth is also important to evaluate for genera of Mithracidae that are currently diagnosed, in part, based on carapace length/width ratios (Windsor & Felder, 2014). The same applies for mithracid species as, for example, allometric growth associated with reproduction was recorded for a modern mithracid species (Cobo & Alves, 2009). Here, we review the fossil record of spider crabs (Majoidea) in the Western Atlantic to elucidate their occurrences through time and their paleobiogeography. Furthermore, various fossil and modern members of the Mithracidae are described or reassigned in the Systematic Paleontology section, and allometric growth of these majoids is discussed because a sufficient number of specimens are available for several species to do so.

## Materials & Methods

We compiled data on all fossil majoid occurrences known from the Western Atlantic (defined here: Argentina to Canada) determined to the genus- and species-levels based on the literature and previously unreported material from the FLMNH Invertebrate Paleontology Collection. Possible majoids not determined to at least the genus-level (e.g., Vega, Feldmann & Sour-Tovar, 1995; Kornecki, 2014; FLMNH IP Collection) were not included.

For the systematics part, the length and width of crab carapaces were measured with digital calipers accurate to 0.03 mm and the ratio between length and width was calculated where possible. Institutional abbreviations for specimens: FSBC: Fish and Wildlife Research Institute, St. Petersburg, Florida, USA; UF: Florida Museum of Natural History at the University of Florida, Gainesville, Florida, USA. Modern UF specimens are housed in Invertebrate Zoology (IZ); fossil specimens in Invertebrate Paleontology (IP).

The electronic version of this article in Portable Document Format (PDF) will represent a published work according to the International Commission on Zoological Nomenclature (ICZN), and hence the new names contained in the electronic version are effectively published under that Code from the electronic edition alone. This published work and the nomenclatural acts it contains have been registered in ZooBank, the online registration system for the ICZN. The ZooBank LSIDs (Life Science Identifiers) can be resolved and the associated information viewed through any standard web browser by appending the LSID to the prefix http://zoobank.org/. The LSID for this publication is: urn:lsid:zoobank.org:pub:6049E531-ABA7-43EA-8308-EEB5029F667F. The online version of this work is archived and available from the following digital repositories: PeerJ, PubMed Central and CLOCKSS.

## Results

### Spider crab distribution in the Western Atlantic

**Figure 1:**
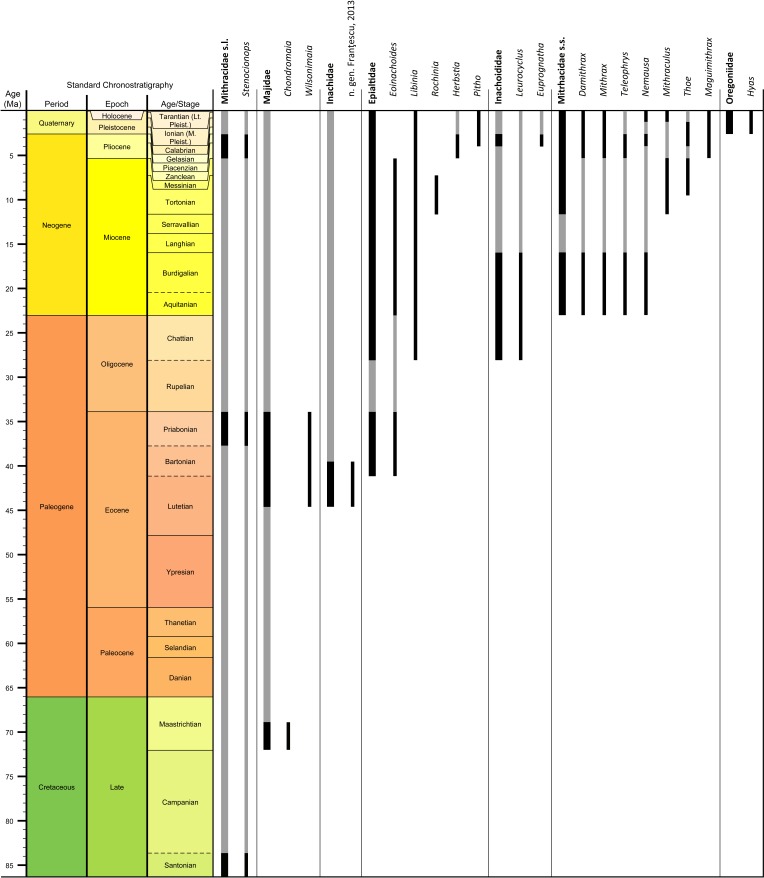
Stratigraphic ranges of all families and genera of spider crabs (Majoidea) in the Western Atlantic based on occurrence data (Table S1). Grey bars represent probable occurrences based on modern or bracketing fossil occurrences for that taxon. Chart arranged stratigraphically and by family. The Hymenosomatidae have no fossil record and the Priscinachidae are only known from Europe thus far. The ranges of families are derived from genera; genus names that were uncertain (aff., ?[genus], or “[genus]”) were not used. Timescale produced with TSCreator 6.4 (http://www.tscreator.org).

**Figure 2:**
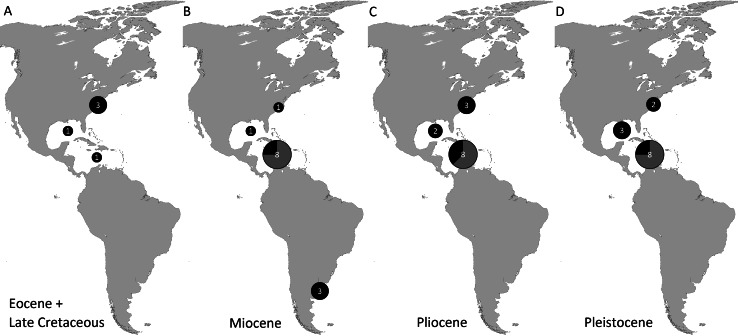
Genus-level diversity of all fossil spider crabs in the Western Atlantic from older to younger epochs (A–D) based on occurrence data (Table S1). Genera of Mithracidae s.s. are indicated as gray parts of pie where present. Genus names that were uncertain (aff., ?[genus], or “[genus]”) were not included. Geographic regions were defined as follows: Atlantic coast North America (Canada to Georgia); Gulf of Mexico (incl. Florida); Caribbean (Cuba to Panama to Barbados); Atlantic coast South America (Guyana and further south). The youngest epoch was arbitrarily chosen for genera that could be either from one epoch or the following. No records are known from the Paleocene and Oligocene (range-through assumption not applied here).

Genera and species known today (15/19 genera or 79%, 14/32 species or 44%) are well-represented in the dataset (Table S1) on fossil spider crabs because most taxon occurrences (>85%) are Neogene and Quaternary in age. Spider crabs in this part of the world first appeared the late Late Cretaceous (Rathbun, 1935; Feldmann et al., 2013), which is younger than the mid-Cretaceous occurrences in Europe (Breton, 2009; Klompmaker, 2013). They become increasingly better represented towards the Recent on the genus- and family-levels (Fig. 1, Tables 1 and S1). For example, two majoid genera are reported from the Late Cretaceous, none from the Paleocene, four from the Eocene, two from the late Oligocene—early Miocene, nine from the Miocene, ten from the Pliocene, and eight from the Pleistocene (Table S1). Applying the range-through assumption (i.e., a genus is present in the entire interval between its first and last known occurrence) yields similar results (Fig. 1): two for the Late Cretaceous, one for the Paleocene, four for the Eocene, two for the Oligocene, four for the late Oligocene—early Miocene, 11 for the Miocene, 13 for the Pliocene, and 14 for the Pleistocene. All modern majoid families are represented except for the Hymenosomatidae, which do not have a fossil record. Most majoid and all mithracid genera are found in the Caribbean region as opposed to in higher latitudes (Fig. 2). Thus far, eight genera are known from the Miocene—Pleistocene deposits from the Caribbean, whereas no more than three genera have been found in all other regions. An analysis of species diversity shows very similar numbers, with an apparent diversity hotspot in the Caribbean (Table 2).

**Table 2:** Species-level diversity of all fossil spider crabs in the Western Atlantic based on occurrence data (Table S1). Species of Mithracidae s.s. are indicated between brackets. The youngest epoch was arbitrarily chosen for species that could be either from one epoch or the following.

	Pleistocene	Pliocene	Miocene	Oligocene	Eocene	Paleocene	Late Cretaceous
Atlantic coast North America (Canada–Georgia)	3	4	1		3		1
Gulf of Mexico Region (incl. Florida)	3 [1]	2	1		1		1
Caribbean Region (Cuba–Panama–Barbados)	9 [7]	8 [5]	9 [7]		1		
Atlantic coast South America (Guyana and further south)			3				

## Discussion

All modern majoid families are represented in Fig. 1 except for the Hymenosomatidae that do not have a fossil record. This is likely related to their small size and weakly calcified exoskeleton (e.g., Ng & Jeng, 1999; Guinot, 2011; Tavares & Santana, 2015; note that Guinot argued that the family does not belong to the Majoidea). Conversely, the Epialtidae and Mithracidae are well-represented, being markedly larger and better calcified, comparatively. Although many gaps exist in the fossil record of Western Atlantic majoids, it becomes clear that most families were present in the Western Atlantic in the Late Cretaceous to Paleogene. Given the oldest occurrences of majoids (Breton, 2009; Klompmaker, 2013), it may be speculated that spider crabs originated in Europe after which they crossed the proto-Atlantic to inhabit the Americas. Conversely, Mithracidae s.s. might have originated in the Western Atlantic because the globally oldest confirmed occurrences thus far are early Miocene in age and from that region (Tables 1 and S1).

Although the pattern that most fossil majoid genera in the Western Atlantic are found in the Caribbean region (Fig. 2) is consistent with the modern latitudinal diversity gradient for decapods, including Brachyura (e.g., Abele, 1982; Steele, 1988), much more research has been done in the (sub)tropical Western Atlantic region and exposures may be more numerous. However, fossil decapods from the eastern coast of the USA have received considerable attention (e.g., Rathbun, 1935; Roberts, 1962; Blow & Manning, 1996; Blow, 2003; Feldmann et al., 2013; Franţescu, 2013). On the other hand, less research has been done on fossil decapods from Brazil and other South American countries south of the Caribbean region thus far (e.g., Aguirre-Urreta, 1990; Casadío et al., 2005; Martins-Neto & Dias Júnior, 2007; Távora, Paixão & Da Silva, 2010). More fossil decapods—including spider crabs—are expected to be present in those regions given the common presence of majoids there today (e.g., Melo, 1996; Melo, 1998; Coelho & Torres, 1990; Bertini, Fransozo & De Melo, 2004; Mantelatto et al., 2004; Alves et al., 2012; Giraldes, Coelho Filho & Smyth, 2015).

The spider crabs *Mithrax arawakum* sp. nov. and *Nemausa windsorae* sp. nov., erected below, add to the number of decapod species known from the lower Miocene limestones at the Duncans Quarry in Jamaica. Portell & Collins (2004) reported on 16 brachyuran decapod species from these limestones, a unique fauna from the Miocene of the Caribbean because 9/14 genera were previously unknown from that region. Moreover, the Duncans Quarry is also of great importance for the knowledge of majoids in the Western Atlantic, yielding four genera and five species thus far, all mithracids. This is half of the majoid genera of the Miocene in the Caribbean region. As is the case for another diverse fossil brachyuran assemblage in the Caribbean, from the Pleistocene of Barbados (Collins & Morris, 1976), the brachyuran fauna from the Duncans Quarry is also associated with corals. This pattern of highly diverse decapod assemblages associated with corals is also observed elsewhere. Cenozoic, coral-associated decapod faunas from Europe are also speciose (e.g., Müller, 1984; Jakobsen & Collins, 1997; Beschin et al., 2007; Gatt & De Angeli, 2010; Beschin, Busulini & Tessier, 2015) as are such decapod faunas from the Mesozoic (e.g., Collins, Fraaye & Jagt, 1995; Fraaije, 2003; Krobicki & Zatoń, 2008; Klompmaker et al., 2013; Klompmaker, Ortiz & Wells, 2013; Robins, Feldmann & Schweitzer, 2013). Moreover, a significant positive correlation exists between global reef abundance and decapod diversity throughout the Mesozoic (Klompmaker et al., 2013). The diverse decapod assemblage from the Duncans Quarry underlines the importance of reefs for the abundance and diversity of decapods in deep time.

## Systematic Paleontology

**Table:** 

Order Decapoda Latreille, 1802–1803
Infraorder Brachyura Linnaeus, 1758
Section Eubrachyura De Saint Laurent, 1980
Superfamily Majoidea Samouelle, 1819
Family Mithracidae MacLeay, 1838

*Maguimithrax* gen. nov.

*Etymology*—Combination of part of the family name of Tobey Maguire, the actor in three Spider-Man movies (2002, 2004, 2007), and *Mithrax*. Gender masculine.

*Type species*—*Maia spinosissimus*Lamarck, 1818, by present designation, gender masculine, extant.

*Species included*—*Maguimithrax spinosissimus* (Lamarck, 1818).

*Material*—UF 12474 (1♀), 11447 (1♂), 11457 (1♀), 31157 (1♂, 1♀), 11388 (1♂, 1♀), all FLMNH IZ collection.

**Figure 3:**
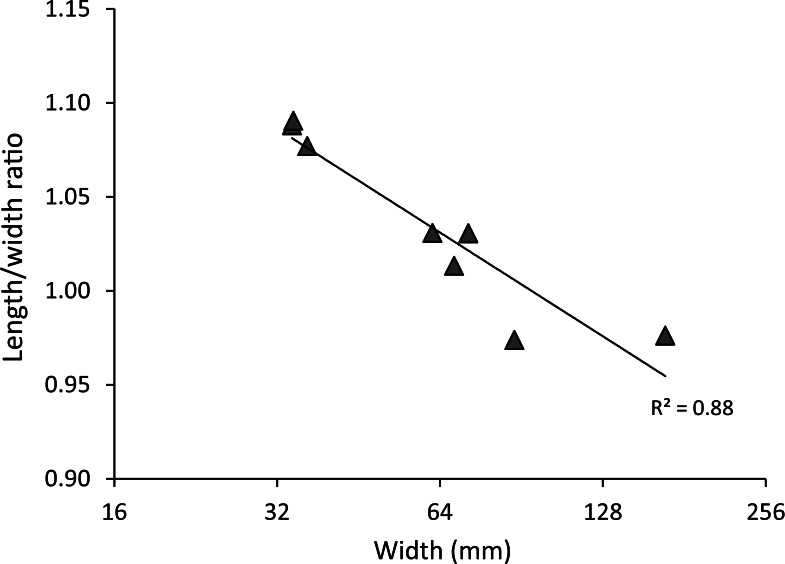
Carapace length/width ratio vs. log_2_ carapace width (mm) for extant *Maguimithrax spinosissimus* (Lamarck, 1818). Maximum length was determined without the rostral spines and width was measured without the anterolateral spines. Trend line is logarithmic (*y* = − 0.08ln(*x*) + 1.3624). Data in Table S2.

*Diagnosis*—Carapace slightly longer than wide to about equally wide as long in large specimens (l/w ratio = ∼ 1.09–0.97) (Fig. 3), maximum reported width without spines 167 mm, rounded to diamond-shaped, without angled transition from antero- to posterolateral margin, covered with spines laterally and tubercles more axially. Upper orbital margin with four to five spines including strong outer orbital spine and axialmost spine; four suborbital spines including two spines on antennal article, axialmost one strongest. Lateral margin bears six spines, anteriormost ones with accessory spines at anterior bases, fifth and sixth spines weaker. Gastric, cardiac, and uro-metagastric regions surrounded by pronounced grooves; other regions less delineated. Chelipeds and other appendages spinose dorsally, less so to smooth ventrally; cheliped propodus with tubercles or spines on upper margins and two to four tubercles on inner side.

**Figure 4:**
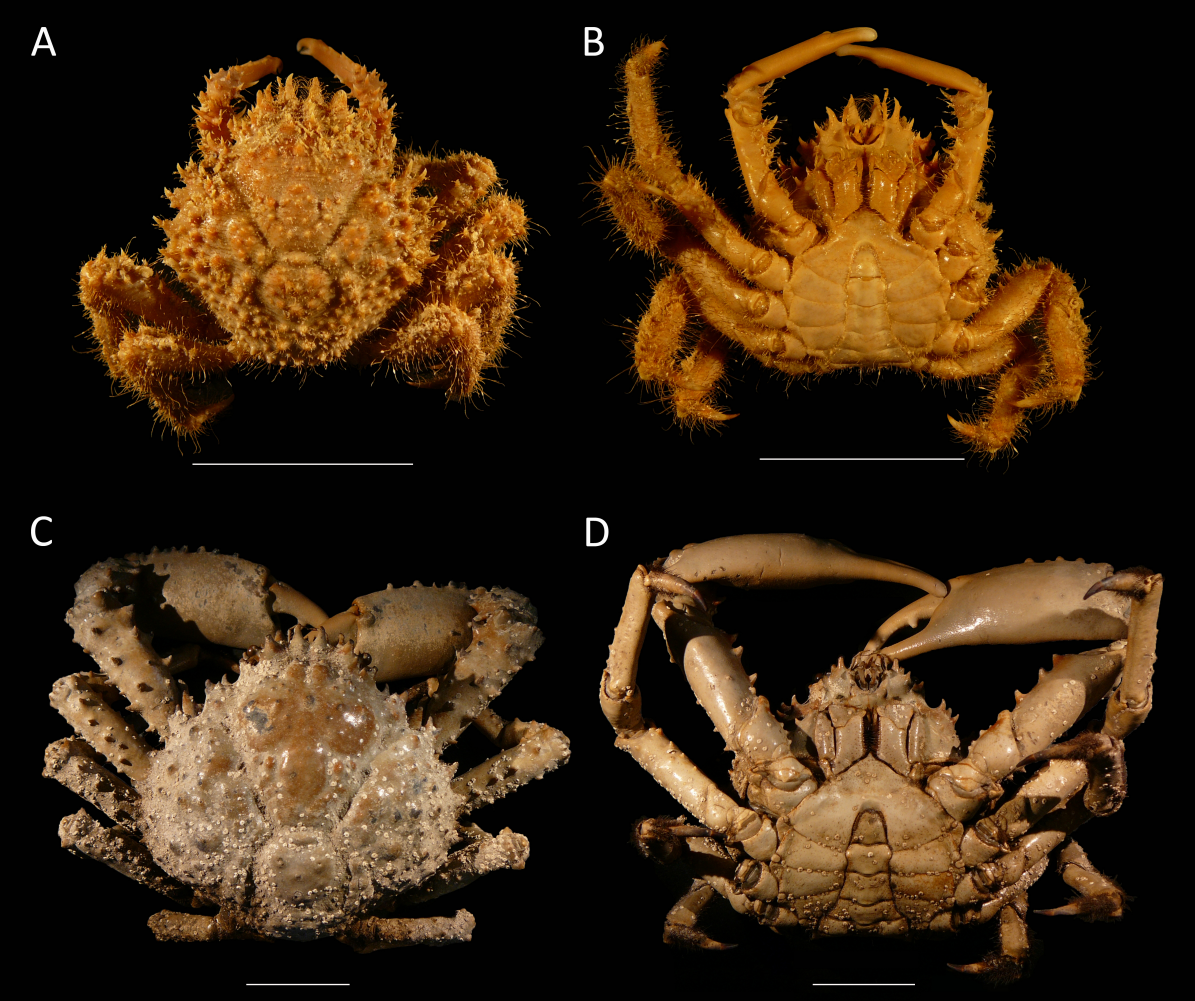
Dorsal and ventral views of modern male specimens of *Maguimithrax spinosissimus* that differ in size. (A, B) UF 11447, Florida, USA; (C, D) UF 11388, Florida, USA (largest specimen). Note the difference in length/width ratios of the carapace. Scale bar width = 30 mm.

*Remarks*—Verrill (1908), Rathbun (1925), and Wagner (1990) all noted that young specimens of *D. spinosissimus* are close to *Nemausa acuticornis* and *N. cornuta*. There are indeed many similarities between *Nemausa* and *D. spinosissimus* including the spinose character of the carapace and appendages, a comparable third maxilliped (see Windsor & Felder, 2014: Fig. 4), a longer than wide carapace in younger individuals (Fig. 4), and a similar groove and region structure of the carapace. Not surprisingly, *D. spinosissimus* has been placed in *Nemausa* (Coelho & Torres, 1990). Several differences exist compared to *Nemausa* as currently defined. The carapace is more rounded to diamond-shaped compared to the pyriform carapaces of *Nemausa* so that the point of maximum width is reached more anteriorly; *D. spinosissimus* bears six lateral spines, whereas *Nemausa* bears five such spines; and the spine at the lateral angle is very strong in *Nemausa* compared to other lateral spines, but it is less prominent than others in *D. spinosissimus*. Molecular phylogenetics support the assertion that *D. spinosissimus* does not fit within *Nemausa* (Windsor & Felder, 2014).

The species has been assigned to *Mithrax* as well (e.g., Provenzano & Brownell, 1977; Wagner, 1990). However, *Mithrax*, as currently defined, is markedly different in that (1) the third maxilliped endopod merus distomesial margin has a deep, angular excavation at the articulation with the palp in *D. spinosissimus*, whereas this merus exhibits no pronounced concavity in *Mithrax* (cf. Windsor & Felder, 2014); (2) the ornamentation of the carapace is more varied in *Mithrax*, consisting of more granules; (3) the propodus bears tubercles and spines in the examined specimens of *D. spinosissimus*, but it is smooth in *Mithrax*; and (4) molecular phylogenetics separates *D. spinosissimus* from *Mithrax* (Windsor & Felder, 2014).

Most recently, the latter authors assigned the species to *Damithrax*. However, it should be noted that *D. spinosissimus* is much more spinose on the dorsal carapace than other species of *Damithrax* (e.g., Desbonne & Schramm, 1867: pl. 8; Rathbun, 1925: pl. 135), including the type species. Moreover, the propodus is not smooth in *D. spinosissimus* unlike in other species of the genus, and specimens across a considerable size range (<75 mm carapace width) are slightly longer than wide or about equally wide as long, unlike the diagnosis of the genus. Not surprisingly, the species plots as a sister taxon to all other modern *Damithrax* spp. (Windsor & Felder, 2014: Fig. 2); the latter authors also indicated that this taxon “is somewhat the outlier” (p. 155). Finally, all three of the discussed genera possess a lateral angle of the carapace, whereas this area is much more rounded in *D. spinosissimus*. Thus, we erect a new genus to accommodate *D. spinossisimus*: *Maguimithrax* gen. nov.

Detailed descriptions of the species and ontogenetic variations were detailed by Rathbun (1925), Williams (1984), and Wagner (1990) that need no repeat here. Sexual dimorphism is evident in that larger males (>∼60 mm carapace width based on the studied material) exhibit a pronounced tooth on the occlusal surface of the dactylus, whereas females do not bear such a tooth.

*Stratigraphic and geographic range*—Extant only, North Carolina—Venezuela (Williams, 1984; Wagner, 1990).

*Damithrax* Windsor & Felder, 2014

*Type species*—*Mithrax pleuracanthus*Stimpson, 1871, extant.

*Species included*—*Damithrax hispidus* (Herbst, 1782–1804) [=*Maia spinicincta* Lamarck, 1818; *Mithrax laevimanus* Desbonne in Desbonne & Schramm, 1867; *Mithrax depressus* Milne-Edwards, 1873–1880 (part); *Mithrax caribbaeus* Rathbun, 1920; *Mithrax carribbaeus*, Ng, Guinot & Davie, 2008 (incorrect spelling)]; *Damithrax pleuracanthus* (Stimpson, 1871); *Damithrax tortugae* (Rathbun, 1920); *Damithrax unguis* (Portell & Collins, 2004).

*Emended diagnosis*—Carapace wider than long [for large specimens, about equally long as wide for small specimens], overall shape pyriform; dorsal surface smooth to tuberculate, not obviously setose; [five] lateral spines or teeth, first two commonly with accessory spine, lateral angle with single spine; posterior margin tuberculate. Rostral horns blunt, sparsely setose, tips not converging, not reaching [far] beyond first movable article of antenna. Antenna fused basal article very broad, forming floor of orbit, bearing two or three blunt marginal spines or teeth, anteriormost the largest, decreasing posteriorly (third often very low, or not developed), anterior two visible in dorsal view. Orbit complete, dorsal margin weakly armed behind strong pre-ocular tooth, eyestalk protected above by single blunt dorsal tooth or tubercle separated by closed fissure from two or three blunt post-ocular teeth or tubercles. Third maxilliped endopod merus distomesial margin deeply, angularly excavated at articulation with palp. Cheliped greater than or equal to carapace length; merus dorsal surface spinous, spines not laminar; carpus varied from smooth to rough; propodus smooth; dactylus with enlarged proximal tooth when mature, opposed margins of fingers otherwise crenulate. Pereiopods two to five (ambulatory legs) decreasing in size anterior to posterior; articles finely setose; merus dorsal surface bearing large tubercles and spines, ventral surface with one to six tubercles or spinules; carpus dorsal surface spinous; propodus without spination; dactylus strong, approximately half length of propodus, dactylar lock well developed (adapted after Windsor & Felder, 2014, changes in brackets).

**Figure 5:**
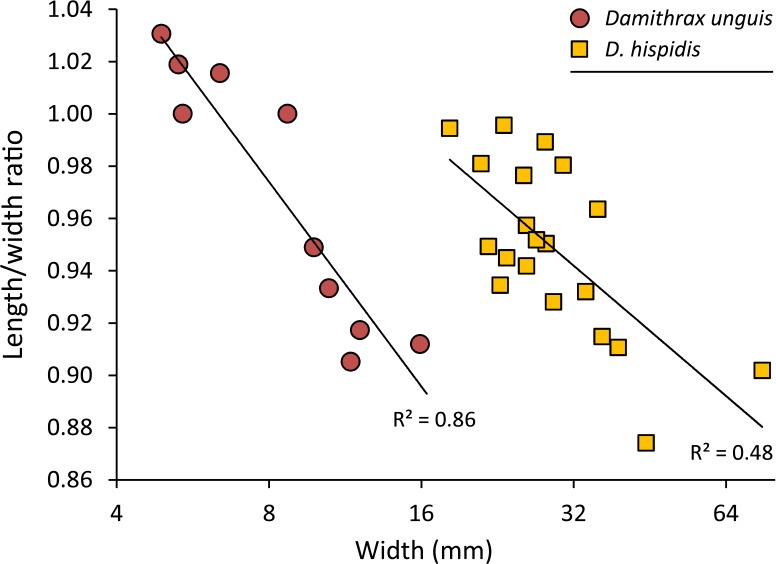
Carapace length/width ratio vs. log_2_ carapace width (mm) for *Damithrax unguis* (Portell & Collins, 2004) from the lower Miocene of Jamaica vs. modern *Damithrax hispidus* (Herbst, 1782–1804) from Florida. Maximum length was determined without the rostral spines and width was measured without the anterolateral spines. Trend lines are logarithmic (*y* = − 0.113ln(*x*) + 1.2088 for *D. unguis*; *y* = − 0.072ln(*x*) + 1.191 for *D. hispidus*). Data in Table S2.

*Remarks*—The diagnosis of Windsor & Felder (2014) mentioned that the carapace is wider than long. While this generally applies to large specimens, small specimens can be about equally long as wide or even slightly longer than wide (Fig. 5).

*Damithrax unguis* (Portell & Collins, 2004)

Figs. 5 and 6

**Figure 6:**
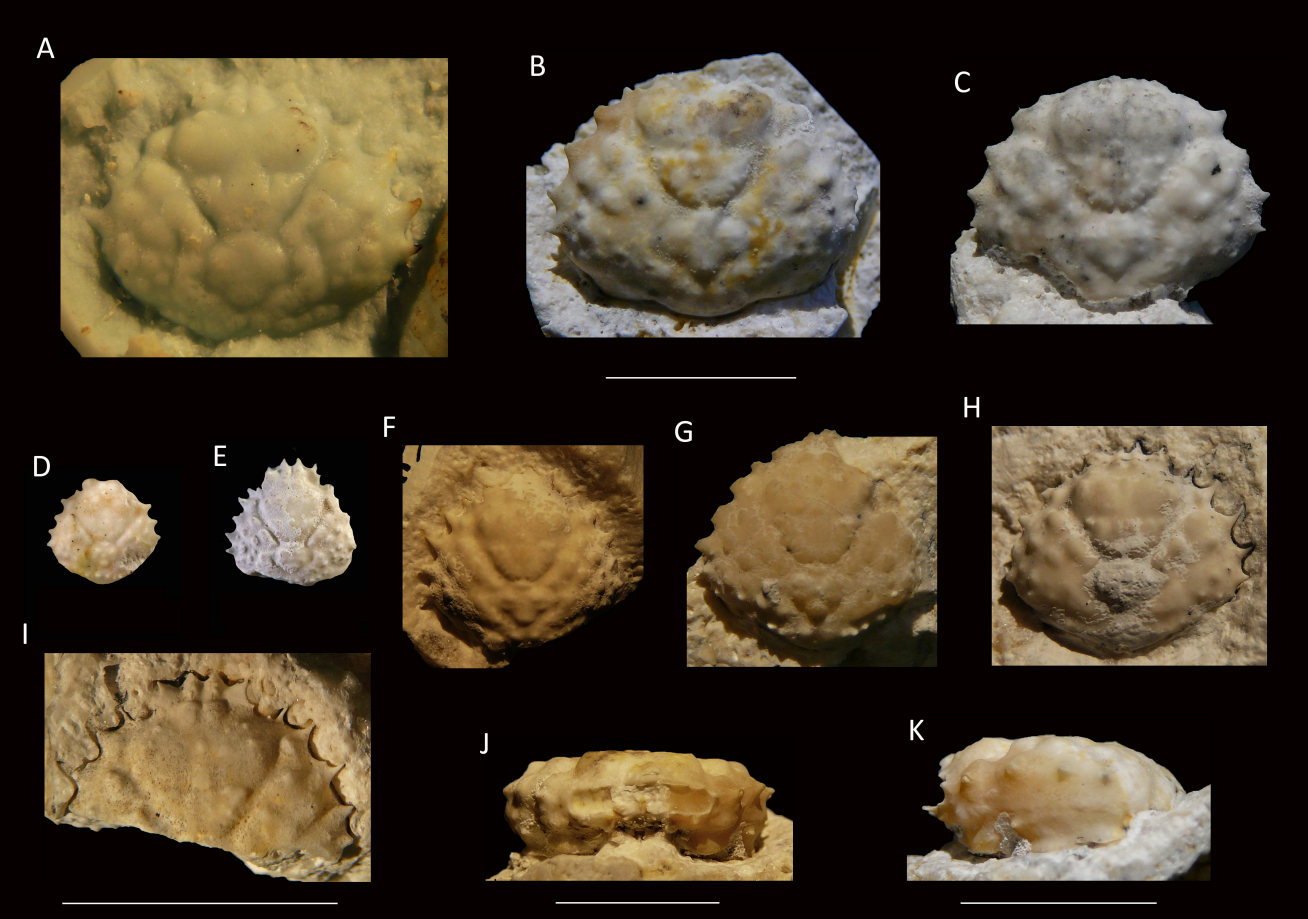
Growth series of dorsal carapaces of *Damithrax unguis* (Portell & Collins, 2004) from the lower Miocene coral-associated limestones of the Montpelier Formation in the Duncans Quarry, Jamaica. (A) = is RTV silicone rubber cast of external mold. (B–K) = internal molds. (A) UF 255051; (B) UF 113677; (C) UF 106768 (paratype); (D) UF 255053; (E) UF 112795; (F) UF 112783; (G) UF 112784; (H) UF 106697 (holotype); (I) UF 103954; (J, K) frontal and left-lateral views of UF 113677. Scale bar below (B) applies to (A–H). Scale bar width = 10.0 mm.

2004 *Mithrax unguis* sp. nov.; Portell & Collins, 2004: p. 117, Fig. 1.6.

*Locality*—FLMNH-IP XJ015: Duncans Quarry 01, Trelawny Parish, Jamaica (18.4710, −77.5796 WGS 84).

*Stratigraphic horizon*—lower Miocene, Montpelier Formation (uppermost unit) (Mitchell, 2004; Portell & Collins, 2004).

*Material*—Holotype: UF 106697; Paratypes: UF 73089, 73165, 103955, 106768, 106772, 111483; Topotypes: UF 112783–112785, 112795, 112942, 112946, 113010, 113011, 113117, 113586, 113587, 113675, 113677, 255051–255054. All internal molds of carapaces, some RTV silicone rubber casts of external molds of carapaces.

*Diagnosis*—Pyriform carapace, l/w ratios vary from ∼0.90 for the largest specimens of ∼16 mm width, to ∼1.00 for small specimens. Short rostrum with two small spines downturned, slightly longer than axialmost inner orbital spine. Four usually single spines (second one may have accessory small spine anteriorly in some specimens) on anterolateral margin excluding outer orbital spine. Forwardly directed shallow orbit with spines on the upper orbital margin: four upper orbital spines including outer orbital spine with center two converging; suborbital margin with three spines, axialmost one strongest. Smaller orbital spines less pronounced in small specimens. Tubercular gastric and branchial regions.

*Description*—See Portell & Collins (2004: p. 117).

*Measurements*— Table S2.

*Remarks*—Portell & Collins (2004) erected *Mithrax unguis* based on early Miocene specimens from the Duncans Quarry, Trelawny Parish, Jamaica. The generic placement was reassessed here because of the revision of extant Mithracidae by Windsor & Felder (2014). Given the close similarity to *Damithrax hispidus*, as was also indicated by Portell & Collins (2004), and a reasonable fit with the current generic diagnosis of *Damithrax*, *Mithrax unguis* is transferred to *Damithrax*. The species cannot be retained in *Mithrax* because of the non-spinose character on the dorsal carapace not including the lateral margins. The species differs from *D. hispidus*, *D. pleuracanthus*, and *D. tortugae* in that the rostrum is sharp instead of blunt and that *D. unguis* seems to have sharper upper orbital spines. Moreover, the length/width ratios separate *D. unguis* from *D. hispidus* (Fig. 5).

Portell & Collins (2004) had a limited number of specimens available and showed measurements for three of them. With additional collecting, preparation, and identification, many new specimens became available allowing for the investigation of ontogenetic variation within the species. Width grows faster relative to the length resulting in a decline of length/width ratios (Figs. 5 and 6). Such allometric growth is especially important for genera of Mithracidae that are currently diagnosed, in part, based on carapace length/width ratios (Windsor & Felder, 2014). For *D. unguis*, one could postulate that width is greater than length for some, width is (sub)equal to length, and even length is greater than width for the smallest specimens. Therefore, providing a range of l/w ratios along with specimen sizes for diagnoses and descriptions seems even more useful.

*Stratigraphic and geographic range*—lower Miocene, Jamaica.

*Damithrax* cf. *pleuracanthus* (Stimpson, 1871)

Figs. 7–10

**Figure 7:**
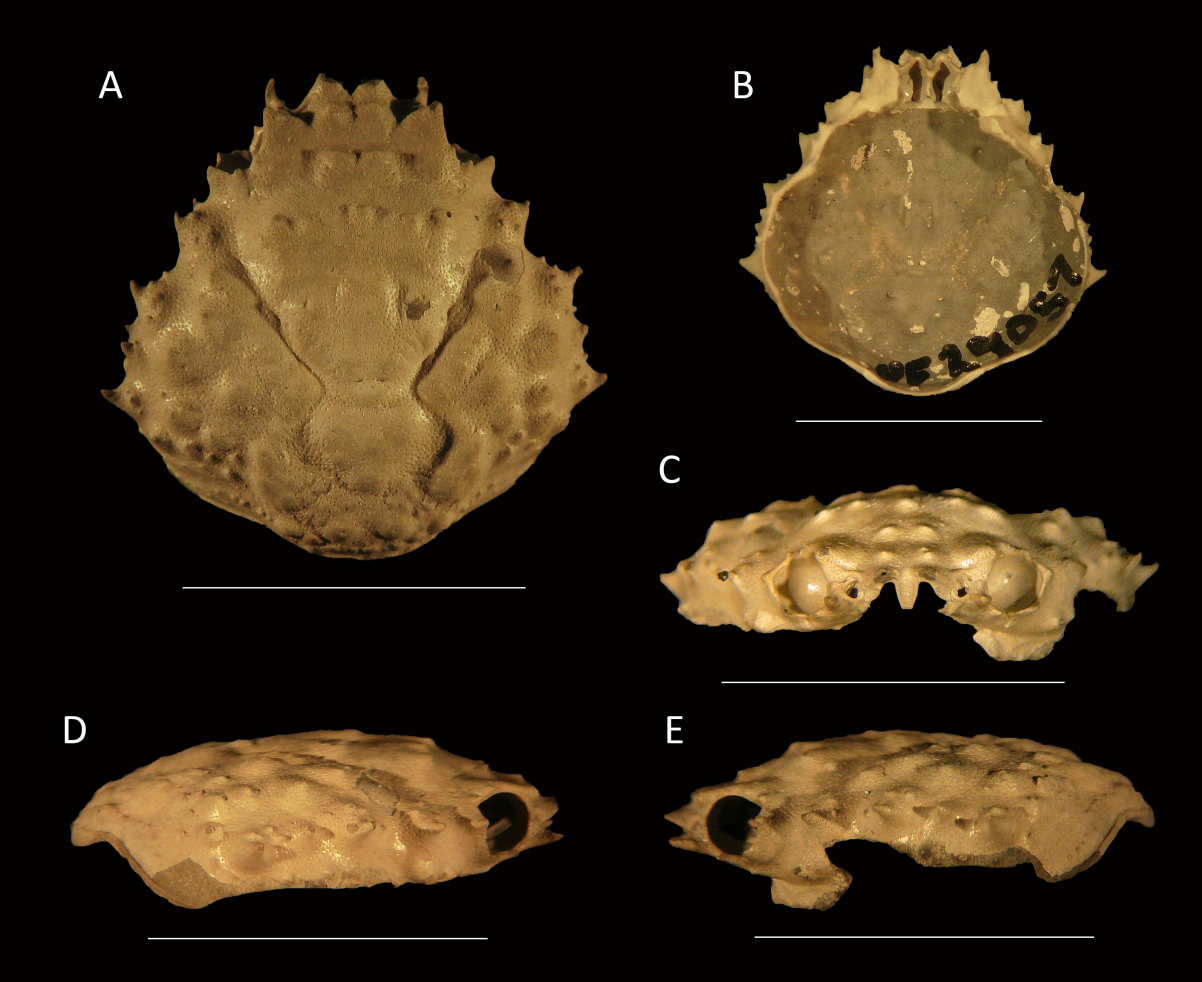
*Damithrax* cf. *pleuracanthus* from the late Pliocene–early Pleistocene of the MacAsphalt Shell Pit, Sarasota County, Florida, USA (UF 29057). (A) Dorsal view; (B) Ventral view; (C) Frontal view; (D) Right-lateral view; (E) Left-lateral view. Scale bar width = 10.0 mm.

**Figure 8:**
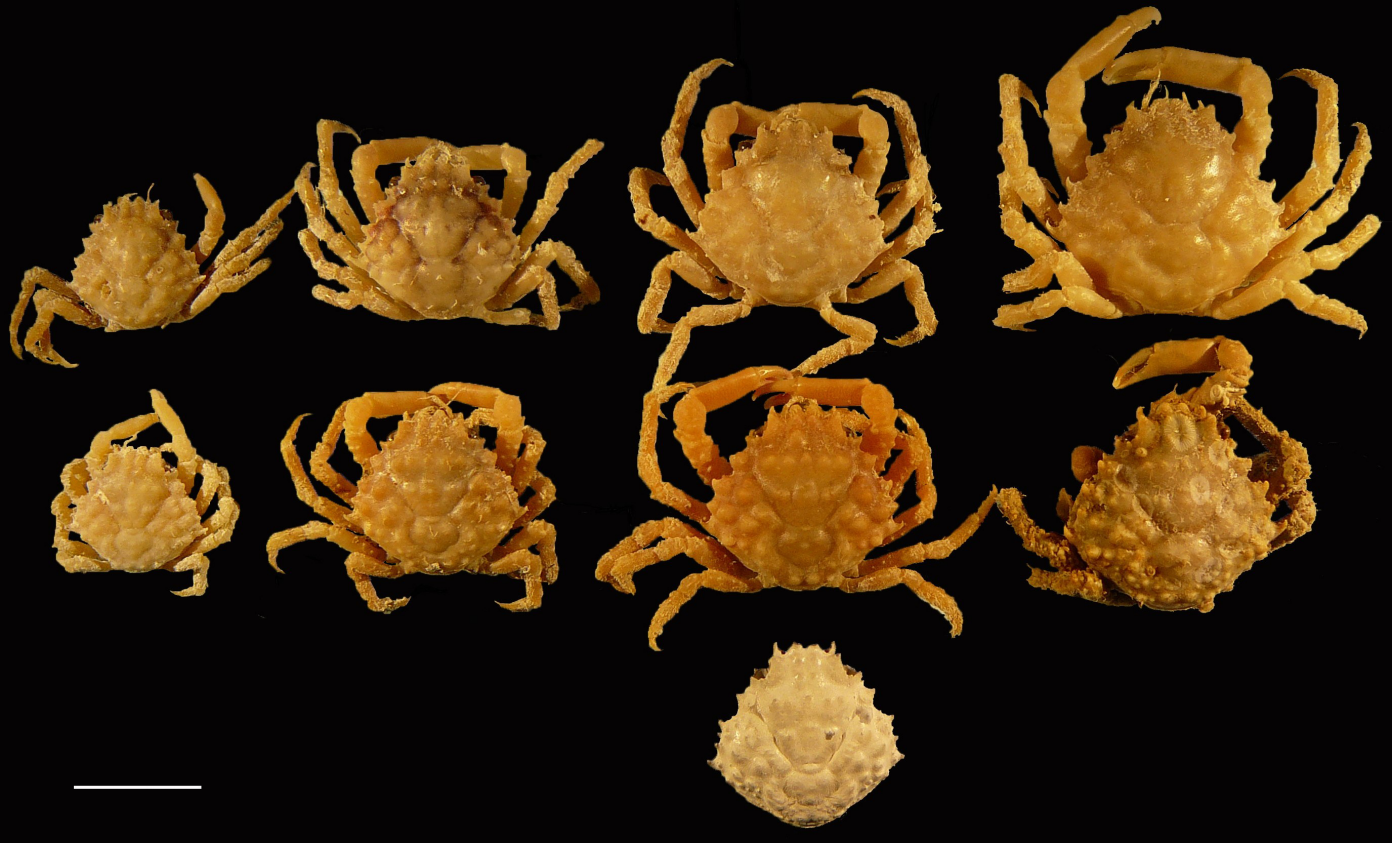
Dorsal views of modern specimens and a single fossil specimen of *Damithrax* spp., all from Florida, USA. Upper row from left to right—modern *D. hispidus*: UF 12475, 11604, 1082, 1086; Middle row—modern *D. pleuracanthus*: UF 3673, 9588 (largest specimen of lot), 7874, 1052; lower row—fossil *Damithrax* cf. *pleuracanthus*: UF 29057. Scale bar width = 10.0 mm.

**Figure 9:**
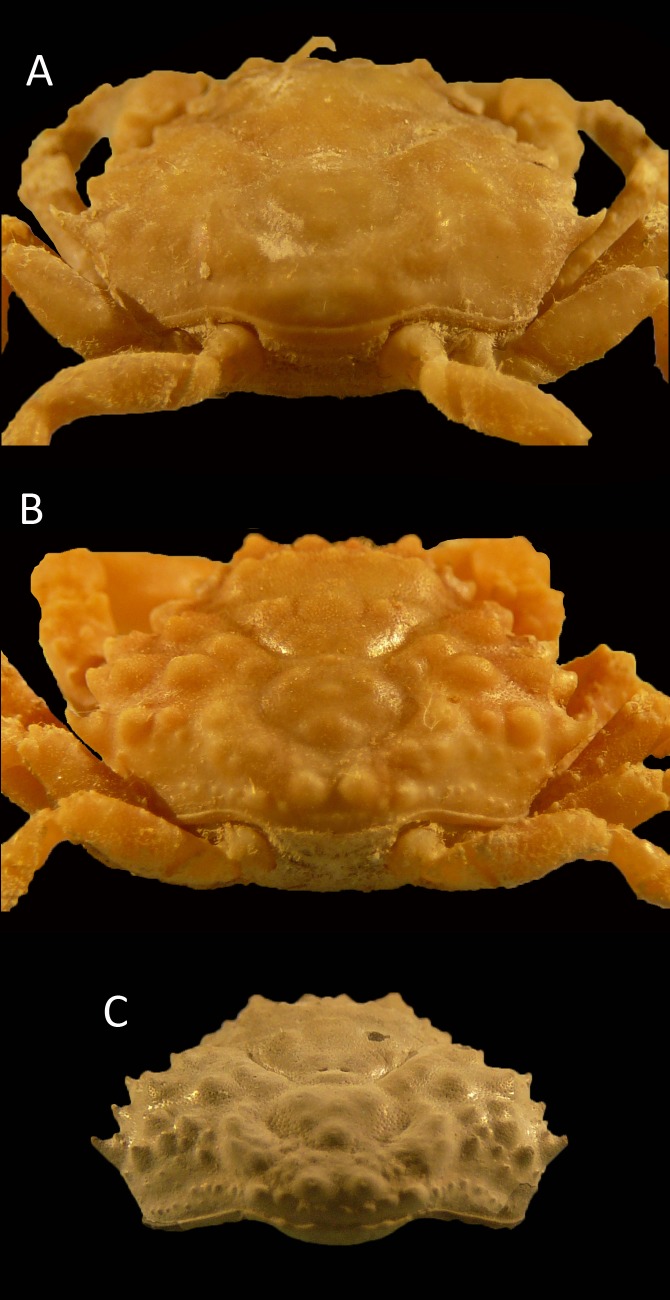
Posterior views of similar-sized, modern specimens and a single fossil specimen of *Damithrax* spp. (A) *D. hispidus*: UF 1082; (B) *D. pleuracanthus*: UF 7874; (C) *Damithrax* cf. *pleuracanthus*: UF 29057. For specimen sizes see Fig. 8.

**Figure 10:**
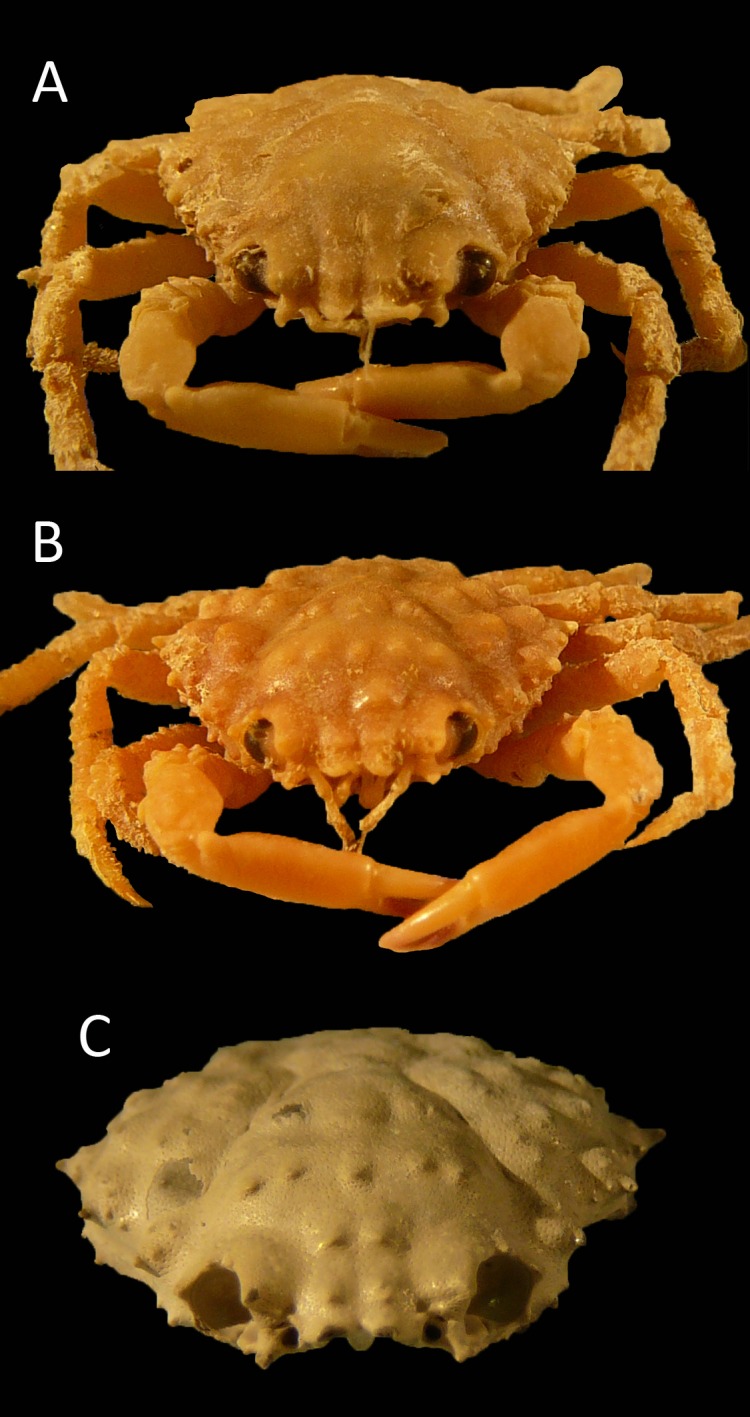
Frontal views of similar-sized, modern specimens and a single fossil specimen of *Damithrax* spp. (A) *D. hispidus*: UF 1082; (B) *D. pleuracanthus*: UF 7874; (C) *Damithrax* cf. *pleuracanthus*: UF 29057. For specimen sizes see Fig. 8.

*Locality*—FLMNH-IP SO001: MacAsphalt Shell Pit, Sarasota County, Florida, USA (27.3666, −82.4520 WGS 84).

*Stratigraphic horizon*—late Pliocene–early Pleistocene, spoil (float).

*Material*—Single carapace (UF 29057), cuticle.

*Diagnosis*—See Williams (1984: p. 334, 335).

*Description*—Carapace pyriform, about as long as wide (l/w ratio = 1.01), maximum width at ∼61% of carapace length, weakly convex longitudinally and moderately so transversely. Rostrum with two forward projections, only bases preserved; with blunt triangular axial projection oriented downward and posteriorly, with rims. Orbits directed anterolaterally, about as wide as tall, deep, with seven spines around orbit: two spines on antennal segment of which the axialmost one is strongest, separated by a notch and then followed by weak spine more laterally; upper orbital margin with four spines including strong outer orbital spine and stronger axialmost spine; two weak spines in between. Circular antennal holes between axialmost suborbital spine and rostral spines. Anterolateral margin with four spines (excluding outer orbital spine), third spine weakest, last spine at transition from antero- to posterolateral margin, oriented laterally. Posterolateral margin more rounded than anterolateral margin, with single small spine just posterior to previous spine. Posterior margin with convex protrusion axially, exhibiting row of tubercles and granules continuing onto posterolateral margin. Frontal region including epigastric region with double row of tubercles. Hepatic regions small, at lower level compared to gastric region, with single anterolateral spine. Protogastric regions bulbous, with major tubercle laterally and less pronounced tubercle axially. Mesogastric region with tubercle on process; base swollen, divided into three regions, central region oval. Uro- and/or protogastric region small, wider than long. Cardiac region pentagonal to triangular, with concave margins, about equally long as wide, tubercular. Branchial regions confluent. Intestinal region not delineated, with two strong tubercles. Cervical groove moderately deep, with two slits axially, V-shaped overall but rounded axially, bends more laterally near anterolateral margin. Shallow groove extends from cervical groove near base hepatic region to below outer orbital spine. Grooves around cardiac and uro- and/or metagastric regions. Dorsal carapace surface of cuticle with very small pits, armed with tubercles all over, more granules posteriorly; row of five tubercles in center of gastric region. Ventrolateral sides below anterolateral margins contain small spines. Of hardened parts: most of ventral surface, abdomen, and appendages lacking.

*Measurements*—Excluding spines and rostrum: 13.9 mm long, 13.8 mm wide.

*Remarks*—The specimen is very well-preserved and is ascribed to *Damithrax* because of the close similarity to extant species, notably *Damithrax hispidus, D. pleuracanthus*, and *D. tortugae*. These modern species were synonymized by Wagner (1990), but Windsor & Felder (2009) resurrected them based on molecular evidence and supported by morphological characters of the appendages. Ornamentation on the dorsal carapace, as was used by Rathbun (1925), was rejected by Windsor & Felder (2009) because of ontogenetic variability (accessory spines and tubercles become more apparent with age), especially within *D. pleuracanthus*. Ontogenetic variability of tubercles on the dorsal carapace was also found for *D. hispidus* in that the largest specimen (75.4 mm carapace width) exhibits fewer tubercles compared to small specimens (<∼30 mm carapace width) (A Klompmaker, pers. obs., 2015). Windsor & Felder (2009) suggested that ornamentation on the merus and carpus of the cheliped can be used to distinguish between *D. hispidus, D. pleuracanthus*, and *D. tortugae*. The FLMNH IZ collection contained sufficient specimens of *D. hispidus* and *D. pleuracanthus* to verify identifications. Indeed, specimens of *D. pleuracanthus* contain more tubercles on the carpus, but ornamental differences were difficult to verify for the merus. While large specimens of *D. hispidus* (>∼35 mm carapace width) often contained two spines on the inner side of the merus, smaller specimens (<∼23 mm carapace width) often contained only a single tubercle, much like similar-sized specimens of *D. pleuracanthus* (Table S3). An additional character to distinguish the two species is ornamentation on the dorsal carapace: tubercles appear better developed on the branchial and gastric regions of *D. pleuracanthus* relative to *D. hispidus* (Figs. 8–10). These differences are confirmed for slightly larger specimens from Rathbun (1925: pls. 146.1, 150.1), whereas *D. tortugae* appears to have even coarser dorsal tubercles (Rathbun, 1925: pl. 147.2). Additionally, a row of small tubercles is present along the posterolateral margin in *D. pleuracanthus*, but is absent in *D. hispidus* for the examined size range (Fig. 9). Thus, we argue that ornamentation on the dorsal carapace can be used to distinguish among modern species for similar-sized specimens. The fossil specimen conforms best to *D. pleuracanthus* in terms of coarseness of the tubercles and the presence of a row of small tubercles along the posterolateral margin. Given the lack of chelipeds to confirm species placement and some minor differences that may represent intraspecific variability (e.g., less robust anterolateral spines in the fossil specimen), the ascription is with some query. Nevertheless, this is the first record of this species in the fossil record. The results in Windsor & Felder (2009) and herein suggest that the ascription of fossil specimens to *D. hispidus* (e.g., Collins & Morris, 1976; Morris, 1993; Collins, Donovan & Dixon, 1996; Varela & Rojas-Consuegra, 2011) may need to be revisited.

*Stratigraphic and geographic range*—late Pliocene–early Pleistocene to Recent, North Carolina—Venezuela—Bermuda (Williams, 1984, see also Tavares & Albuquerque, 1993).

*Mithrax* Milne-Edwards, 1873–1880

*Type species*—*Cancer aculeatus*Herbst, 1782–1804 (see Windsor & Felder, 2014), extant.

*Species included*—*Mithrax aculeatus* (Herbst, 1782–1804) [=*Cancer spinosus* (Herbst, 1782–1804); *Cancer aculeatus* Fabricius, 1793; *Mithrax pilosus* Rathbun, 1892; *Mithrax verrucosus* Milne-Edwards, 1832; *Mithrax plumosus* Rathbun, 1901; *Mithrax trispinosus* Kingsley, 1879]; *Mithrax armatus* De Saussure, 1853 [=*Mithrax orcutti* Rathbun, 1925]; *Mithrax arawakum* sp. nov.; *Mithrax bellii* Gerstaecker, 1857; *Mithrax besnardi* Melo, 1990; *Mithrax braziliensis* Rathbun, 1892; *Mithrax caboverdianus* Türkay, 1986; *Mithrax clarionensis* Garth, 1940; *Mithrax hemphilli* Rathbun, 1892; *Mithrax leucomelas* Desbonne in Desbonne & Schramm, 1867; *Mithrax tuberculatus* Stimpson, 1860.

*Diagnosis*—See Windsor & Felder (2014: p. 162, 163).

*Mithrax arawakum* sp. nov.

Fig. 11

**Figure 11:**
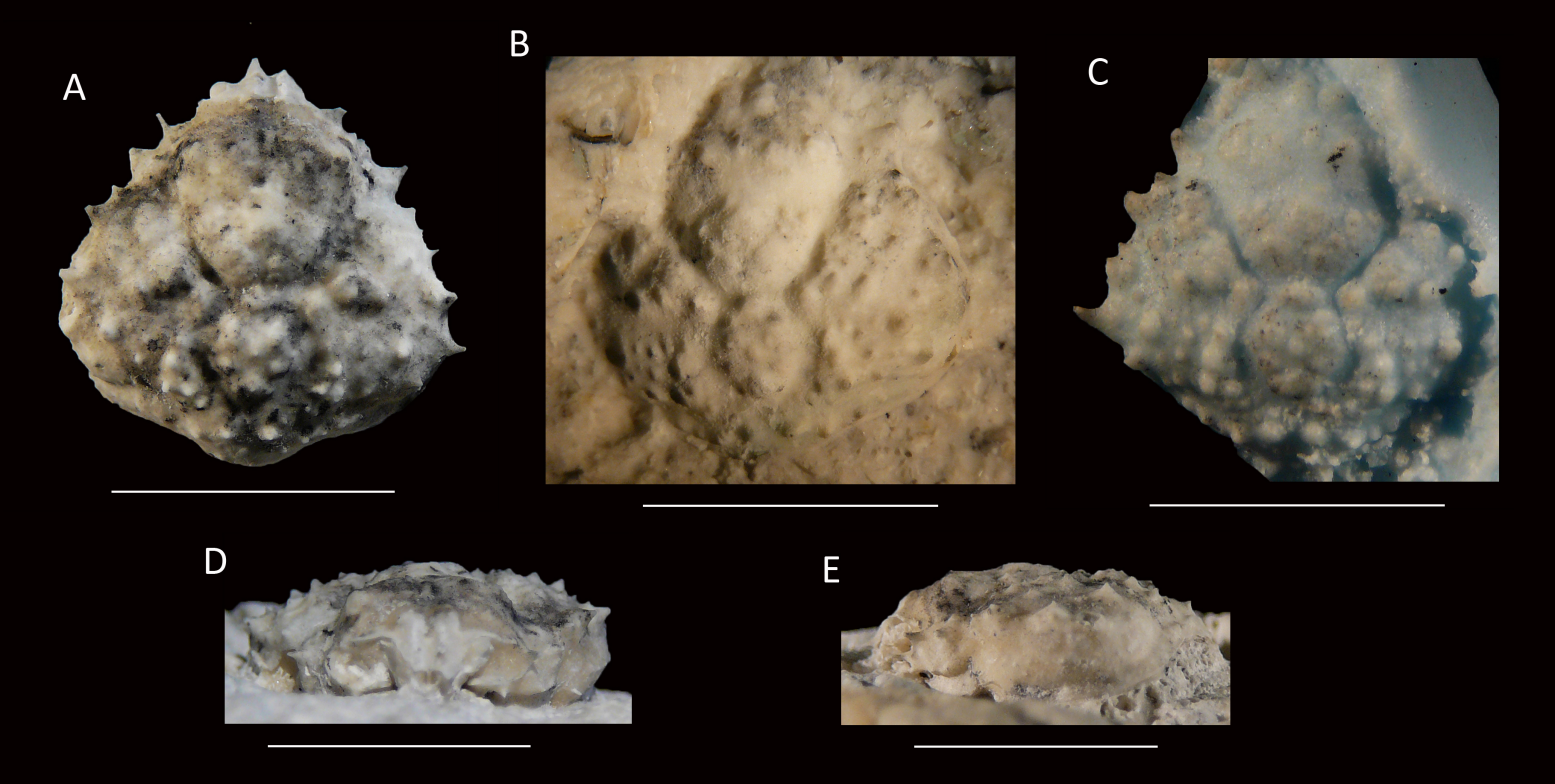
Type specimens of *Mithrax arawakum* sp. nov. from the lower Miocene coral-associated limestones of the Montpelier Formation in the Duncans Quarry, Jamaica. (A, D, E) Holotype, UF 112682, in dorsal, frontal, and left-lateral views, resp.; (B) Paratype, external mold, UF 112941; (C) Paratype, cast of external mold, UF 112941. Scale bar width = 10.0 mm.

*Etymology*—Named in honor of the Arawak natives, who settled the island of Xaymaca (Jamaica).

*Type material*—UF 112682 (holotype, internal mold), UF 112941 (paratype, external mold + RTV silicone rubber cast).

*Type locality*—FLMNH-IP XJ015: Duncans Quarry 01, Trelawny Parish, Jamaica (18.4710, −77.5796 WGS 84).

*Type horizon*—lower Miocene, Montpelier Formation (uppermost unit) (Mitchell, 2004; Portell & Collins, 2004).

*Material*—No material known other than type specimens.

*Diagnosis*—Carapace pyriform, slightly longer than wide (l/w ratio = 1.03 for holotype). Short rostrum with two small spines downturned. Orbits directed forward, with at least four distinct spines around orbit: one long spine at angle of suborbital margin near rostral horns, other such spines not preserved; a slender and long outer orbital spine; a small central upper orbital spine; and a large projection on upper margin near rostral horns. Anterolateral margin with four strong spines (excluding outer orbital spine), middle two with small spine at anterior base; last spine at transition from antero- to posterolateral margin, oriented laterally. Posterolateral margin more rounded than anterolateral margin, with single small spine just posterior to previous spine. Frontal region with two longitudinal rims connecting to rostral spines and tubercular epigastric regions. Cervical groove deep and wide, U-shaped. Branchiocardiac groove strongest around cardiac region, weaker more laterally. Dorsal carapace surface armed with tubercles, granules, and spines (especially on branchial regions), not very densely so.

*Description*—Carapace pyriform, slightly longer than wide (l/w ratio = 1.03 for holotype), maximum width at ∼65% of carapace length, weakly convex longitudinally and moderately so transversely. Short rostrum with two small spines downturned. Orbits directed forward, wider than tall, not very deep, at least four distinct spines around orbit: one long spine with a smaller spine axially at angle of suborbital margin near rostral horns, other such spines not preserved (may be broken); a slender and long outer orbital spine; a small central upper orbital spine; and a large projection on upper margin near rostral horns. Single small spine present below orbit. Anterolateral margin with four strong spines (excluding outer orbital spine), middle two with small spine at anterior base; last spine at transition from antero- to posterolateral margin, oriented laterally. Posterolateral margin with single small spine just posterior to previous spine. Posterior margin with convex protrusion axially, with row of granules adjacent to convexity. Frontal region with two longitudinal rims connecting to rostral spines and tubercular epigastric regions. Hepatic regions small, at lower level compared to gastric region, with single strong anterolateral spine. Protogastric regions bulbous, with major tubercle laterally and less pronounced one axially. Mesogastric region with tubercle on process; base swollen, divided into three regions. Uro- and/or protogastric region small, appears as a laterally elongated tubercle. Cardiac region pentagonal, about equally long as wide, tubercular. Branchial regions weakly divided; epi- and mesobranchial regions confluent, tubercular; metabranchial separated from others, with spines, tubercles, and granules. Intestinal region not delineated, with two strong tubercles. Cervical groove deep and wide, U-shaped, bends more laterally near anterolateral margin to continue on ventral carapace, where it bends forward. Short groove extends from cervical groove near base hepatic region to outer orbital spine. Branchiocardiac groove strongest around cardiac region, weaker more laterally, not expressed to very weak on ventral carapace. Dorsal carapace surface armed with tubercles, granules, and spines (especially on branchial regions), not very densely so; row of five tubercles in center of gastric region. Of hardened parts: most of ventral surface, abdomen, cuticle, and appendages lacking.

*Measurements*—Excluding spines and rostrum: 14.0 mm long, 13.6 mm wide (UF 112682); length not measurable, 13.0 mm wide (UF 112941).

*Remarks*—The species appears to fit best in *Mithrax* because (1) the carapace is about equally long as wide (l/w ratio = 1.03); (2) the dorsal ornamentation has tubercles, granules, and spines (although less obvious than in most *Mithrax* spp.); and (3) the orbit is weakly produced and has two spines on the upper margin excluding the outer orbital spine.

The new species differs from all other congenerics. The carapaces of *M. aculeatus*, *M. armatus, M. bellii*, *M. besnardi*, and *M. hemphilli* exhibit a dense cover of granules (Rathbun, 1925: pls. 138.3, 139, 140, 142, 144; Garth, 1946: pl. 66, 1958: pl. 40.2; Melo, 1990; A Klompmaker, pers. obs., 2015 FLMNH IZ collection for *M. aculeatus*), whereas granules are much less abundant in the new species. Additionally, *M. besnardi* has a higher number of spines on the upper orbital margin (four excluding outer orbital spine instead of two). For *M. braziliensis*, Rathbun (1892) mentioned that the regions of this species are weakly defined, unlike the present species. Moreover, the upper orbital margin bears two small spines, whereas the new species bears one small and one larger one excluding the outer orbital spine. Although the ornamentation on the dorsal carapace of *M. caboverdianus* seems comparable (tubercles and spines with some interspersed granules) to the new species, the similar-sized holotype in Türkay (1986) (15.3 mm long) appears somewhat longer than wide (l/w ratio = 1.09) relatively (1.03 for *Mithrax arawakum* sp. nov.), but more specimens are needed to confirm this potential difference. Distinct rostral spines are missing in *M. caboverdianus*, but are present in *Mithrax arawakum* sp. nov. Additionally, the cardiac region in *M. caboverdianus* appears wider. The upper orbital margin contains more spines in *M*. *clarionensis* and the spines on the lateral margin are less prominent for a similar-sized specimen (Garth, 1940: pl. 15). *Mithrax leucomelas* was never figured and the specimen was already lost when Desbonne & Schramm (1867) erected the species. The description suggests that this species is different from the new species because *M. leucomelas* is said not to be spinose, the anterolateral margins are only slightly toothed, and the lateral angle does not bear a spine, unlike the specimens herein. Lastly, the new species is less tubercular than *M. tuberculatus* for a similar-sized specimen (Rathbun, 1925: pl. 151.1). Moreover, the rostral horns of *M. tuberculatus* are blunt; they are sharp in the new species.

This taxon is of special importance because it constitutes the oldest confirmed record of fossil *Mithrax*. The early Miocene record of *Mithrax* sp. from Cuba (Varela, 2013) is based on a fixed finger, which may not be sufficient for a genus ascription in light of the recent revision (Windsor & Felder, 2014). The same applies to other appendage fragments attributed to *Mithrax* sp. as well as incomplete carapaces (see Table S1).

The holotype is an internal mold, whereas the paratype is an external mold. Since the size of the two specimens is similar, the ornamentation can be compared. The cast of the external mold shows ornamentation that is largely the same to that of the internal mold, but some granules appear larger (those near the posterior margin).

*Stratigraphic and geographic range*—lower Miocene, Jamaica.

*Nemausa* Milne-Edwards, 1873–1880

*Type species*—*Pisa spinipes*Bell, 1836, subsequent designation, extant.

*Species included*—*Nemausa acuticornis* (Stimpson, 1871); *Nemausa cornuta* (De Saussure, 1857) [=*Nemausa rostrata* Milne-Edwards, 1873–1880]; *Nemausa donovani* (Portell & Collins, 2004); *Nemausa windsorae* sp. nov.; *Nemausa sinensis* (Rathbun, 1892); *Nemausa spinipes* (Bell, 1836) [=*Mithrax mexicanus* Glassell, 1936].

*Diagnosis*—See Windsor & Felder (2014: p. 163, 164), but note that the now included fossil species and *N. sinensis* all have a tubercular rather than spinous character on the dorsal surface.

**Figure 12:**
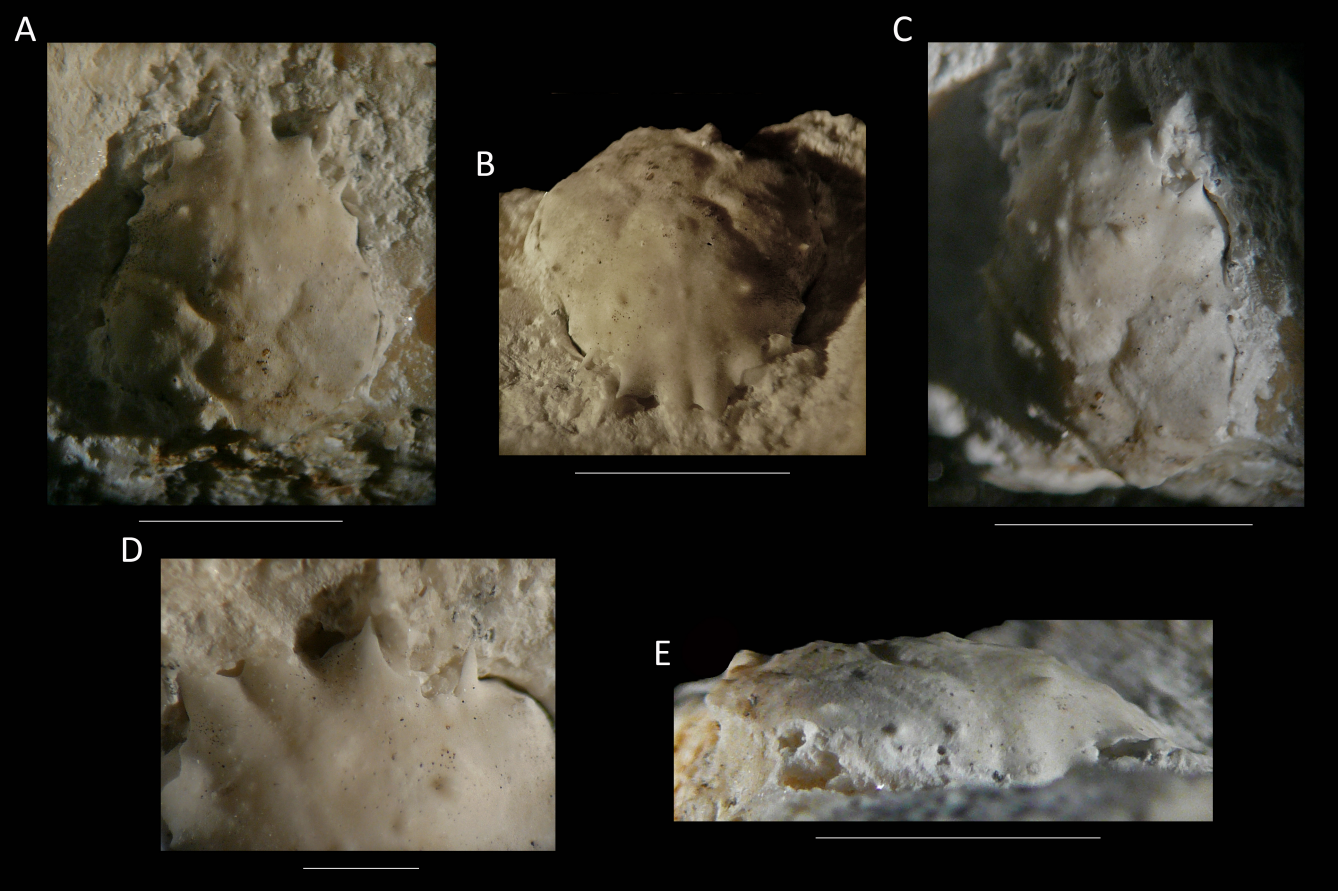
The holotype of *Nemausa donovani* (Portell & Collins, 2004) from the lower Miocene coral-associated limestones of the Montpelier Formation in the Duncans Quarry, Jamaica (UF 103958). (A) Dorsal view; (B) frontal view; (C) angled right-lateral view; (D) upper view of rostrum and orbit; (E) right-lateral view. Scale bar width = 5.0 mm for (A–C, E); 1.5 mm for (D).

*Remarks*—*Mithrax donovani* (Fig. 12) is moved to *Nemausa* because the carapace is longer than wide in *Nemausa*, whereas the carapace length is subequal to the width or wider than long in the diagnosis of *Mithrax* (see Windsor & Felder, 2014). The small size of the specimen (6.7 mm maximum width, 8.0 mm preserved length excluding rostrum) suggests that not all characters may have fully developed yet (anterolateral spines, dorsal ornamentation, length/width trajectory), so the ascription to this genus is preliminary until better preserved material is discovered.

**Figure 13:**
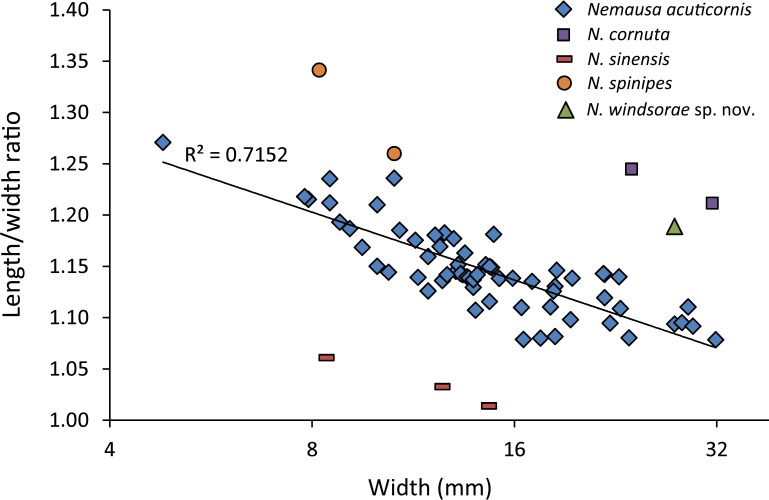
Carapace length/width ratio vs. log_2_ carapace width (mm) for *Nemausa* spp. *Nemausa donovani* was not included because the total length could not be determined. Maximum length was determined without the rostral spines and width was measured without the anterolateral spines. Trend line is logarithmic (*y* = − 0.095ln(*x*) + 1.4013). Data in Table S2.

As for other spider crabs studied herein, ontogenetic change in the length/width ratios is evident for *Nemausa* as well (Fig. 13). The relationship for the species with the most specimens available, *N. acuticornis*, is best explained by a logarithmic trend line, suggesting that length/width ratios change faster in smaller specimens.

*Nemausa windsorae* sp. nov.

Figs. 13 and 14

**Figure 14:**
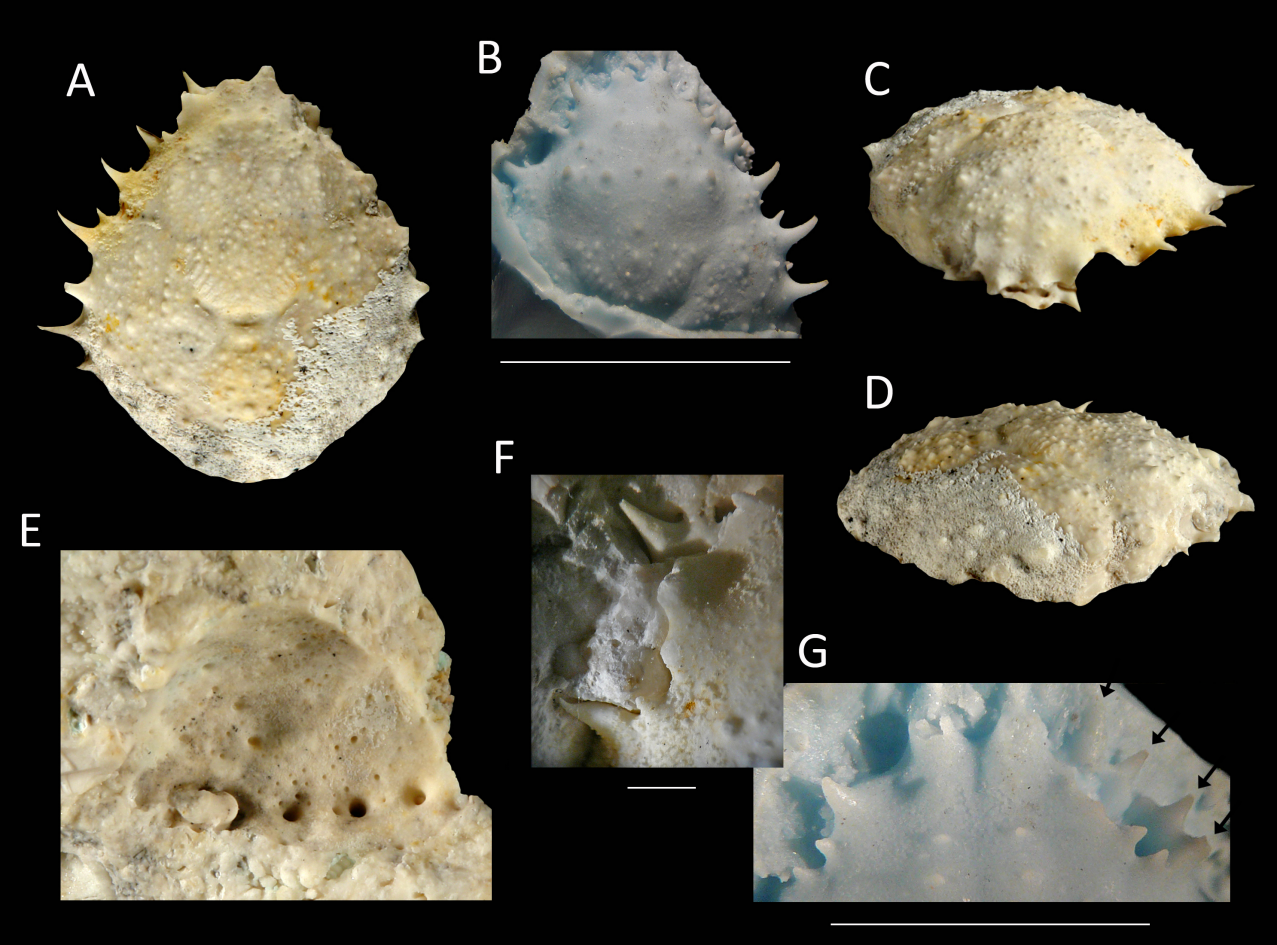
The holotype of *Nemausa windsorae* sp. nov. from the lower Miocene coral-associated limestones of the Montpelier Formation in the Duncans Quarry, Jamaica (UF 113651). (A) Dorsal view (internal mold); (B) dorsal view (cast of external mold); (C) frontal view; (D) right-lateral view; (E) external mold; (F) upper margin left orbit; (G) cast showing bases of rostral horns and various orbital spines in more detail. Arrows in (G) indicate suborbital spines and broken outer orbital spine. Scale bar width = 20 mm for (A–E); 2.0 mm for (F); 10 mm for (G).

*Etymology*—Named after Amanda M. Windsor for her work on extant majoids, especially mithracids.

*Type material*—Holotype and sole specimen, UF 113651 (internal mold with some cuticle, external mold + RTV silicone rubber cast).

*Type locality*—FLMNH-IP XJ015: Duncans Quarry 01, Trelawny Parish, Jamaica (18.4710, −77.5796 WGS 84).

*Type horizon*—lower Miocene, Montpelier Formation (uppermost unit) (Mitchell, 2004; Portell & Collins, 2004).

*Material*—No material known other than type specimen.

*Diagnosis*—Length/width ratio pyriform carapace = 1.19; orbital margins with seven spines, one long spine at angle of suborbital margin near rostral horns and two additional, smaller spines on same margin; anterolateral margin of carapace with four strong spines, anteriormost two with small spine at anterior base; mesogastric region flattened, anterior part not defined.

*Description*—Carapace pyriform, length/width ratio = 1.19, maximum width at 59% of carapace length, moderately convex longitudinally and transversely. Rostrum incompletely preserved, but with bases of two diverging spines. Orbits anterolaterally directed, wider than tall, deepest in most lateral part, seven spines around orbit: one long spine at angle of suborbital margin near rostral horns and two additional, smaller spines on same margin, separated by notch that marks boundary between antennal segment and rest of suborbital structure; one strong outer orbital spine with elongated base; three supraorbital spines, one closest to rostrum strongest. Anterolateral margin with four strong spines (excluding outer orbital spine), anteriormost two with small spine at anterior base; last strong spine at transition from antero- to posterolateral margin, directed laterally. Posterolateral margin more rounded than anterolateral margin, with single spine just posterior to previous spine. Gastric and hepatic regions mostly undifferentiated; epigastric regions appear as tubercles; base of mesogastric region swollen, anterior part not defined; uro- and/or metagastric region small, wider than long, sandwiched between mesogastric and cardiac regions. Cardiac region hexagonal. Branchial and intestinal regions confluent. Cervical groove deepest axially; curves around base mesogastric region, then becomes shallower and bends transversely to intersect lateral margin between first and second anterolateral spines. Branchiocardiac groove only defines lateral parts of cardiac region, does not reach lateral margin. Dorsal carapace surface armed with larger and smaller tubercles; row of five pronounced tubercles in center of gastric region; other strong tubercles present on epigastric, branchial, and cardiac regions. Of hardened parts: ventral surface, abdomen, and appendages missing; rostral spines largely missing.

*Measurements*—Excluding spines and rostrum: 27.7 mm long, 23.3 mm wide, and 14 mm tall (as preserved).

*Remarks*—The anterolateral spines are about equally prominent on the cast and the internal mold. The bases of the rostral spines and many of the orbital spines are much better seen on the cast. This is not surprising given the delicate nature of spines, which have the tendency to break easily on the internal mold. Perhaps surprisingly, the small tubercles on the dorsal carapace are not as numerous on the cast, yet another example that ornamentation with and without the cuticle can differ (see Lörenthey & Beurlen, 1929; Klompmaker, Hyžný & Jakobsen, 2015). Here, the difference can at least in part be explained by the fact that still some cuticle is present near/in those tubercles in the external mold, leading to the absence or less obvious tubercles on the cast.

**Figure 15:**
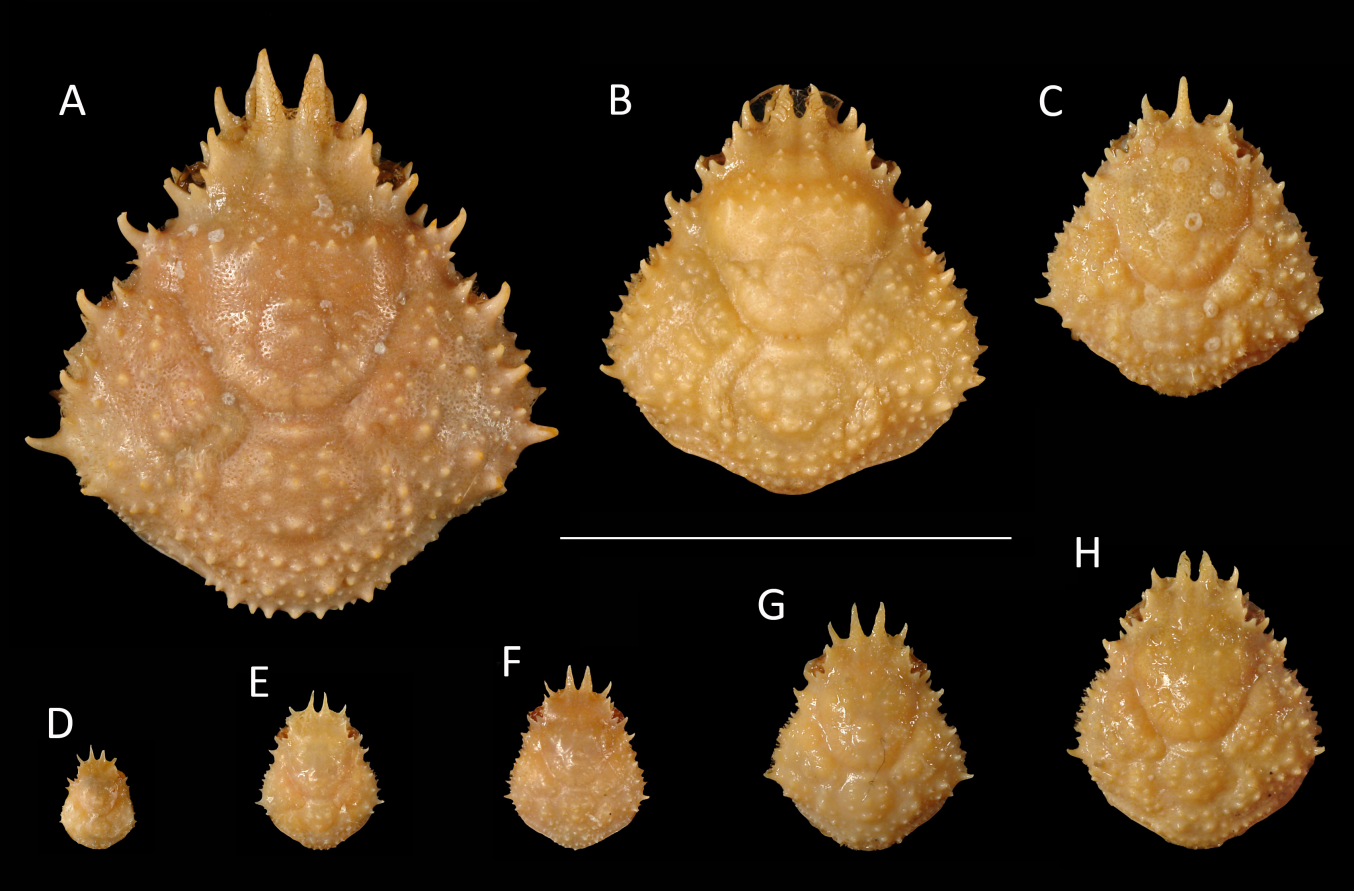
Growth series of dorsal carapaces of modern *Nemausa acuticornis* (Stimpson, 1871) from various localities of the Atlantic coast of Florida, USA. Note that specimens become relatively wider with age. (A) FSBC I-9758; (B) FSBC I-050561; (C) FSBC I-050562 (note the ‘unicorn’ rostrum instead of a double-horned rostrum); (D) FSBC I-050562; (E) FSBC I-050562; (F) FSBC I-050561; (G) FSBC I-050562; (H) FSBC I-050562. Scale bar width = 30 mm.

*Nemausa acuticornis* is consistently more differentiated in the gastric region (e.g., center mesogastric region better defined and outlined: Fig. 14; Rathbun, 1925: pl. 136.1; Felder et al., 2014: Fig. 7C). Moreover, as mentioned by Rathbun (1925: p. 391), Fig. 15 shows that the suborbital margin of *N. acuticornis* contains only one pronounced spine between the outer orbital spine and the spines on the antennal segment, whereas this specimen bears two distinct spines there. Finally, *N. acuticornis* is relatively wider for specimens of the same size (Fig. 13).

*Nemausa cornutus* exhibits more spinose ornamentation on the carapace (Rathbun, 1925: pl. 137.3 and 137.4) even though the specimens are larger (larger specimens tend to have weaker ornamentation compared to younger specimens from the same species in the Mithracidae). Moreover, the specimens in Rathbun (1925: pl. 137.3 and 137.4) are narrower (Fig. 13), although more specimens are needed to statistically test this difference.

*Nemausa sinensis* has a lower l/w ratio (1.03 (Garth, 1958: pl. 41.1), 1.06 (Rathbun, 1892: pl. 38.2)) compared to *N. windsorae* sp. nov. (1.19) (Fig. 13). Furthermore, stronger tubercles are present on *N. sinensis*.

Very few specimens of *N. spinipes* are figured, with Rathbun (1925) showing the best image. *Nemausa spinipes* has a better defined mesogastric region (Rathbun, 1925: pl. 136.4) and all anterolateral spines are single and not associated with smaller spines as in the specimen under study. The same author also showed a very strong tubercle on the posterior part of the mesogastric region, not seen in the specimen under study; and two instead of one tubercle are present around the location where the mesogastric process would be.

*Nemausa donovani* is different in that the mesogastric region is outlined entirely and a distinct elevation is seen in the center of the posterior part of this region, both unlike in the new species. This is unlikely to be related to ontogeny because the mesogastric features appear stable throughout ontogeny in a congeneric species (Fig. 15). Although anterolateral spines become more prominent throughout ontogeny in *Nemausa* (*N. acuticornis*, Fig. 15), the difference between *N. donovani* and *N*. *windsorae* sp. nov. is much greater, supporting the hypothesis that these are two separate species. Furthermore, *N. windsorae* sp. nov. bears a denser ornamentation of tubercles, which may only in part be explained by ontogeny (Fig. 15) because even the smallest specimen of *N. acuticornis* bears distinct tubercles on the branchial regions, whereas these regions are nearly smooth in *N. donovani*, unlike for *N. windsorae* sp. nov.

*Stratigraphic and geographic range*—lower Miocene, Jamaica.

## Supplemental Information

10.7717/peerj.1301/supp-1 Table S1 Database fossil majoids from the Western Atlantic.

10.7717/peerj.1301/supp-2 Table S2 Measurements modern and fossil mithracids from the Western Atlantic.

10.7717/peerj.1301/supp-3 Table S3 Data on *Damithrax hispidus* from Florida.

## Additional Information and Declarations

### New Taxa Registration

The following information was supplied regarding the registration of a newly described species: Zoobank: *Maguimithrax* gen. nov.: http://zoobank.org/NomenclaturalActs/BE0D3F8C-7FD8-43A8-A42A-DE797B31A58E *Mithrax arawakum* sp. nov.: http://zoobank.org/NomenclaturalActs/971193E9-B5BA-44EA-A889-C11B01B6C785 *Nemausa windsorae* sp. nov.: http://zoobank.org/NomenclaturalActs/2A8E6FEB-E453-4FFE-948B-237AE523652F.

## References

[ref-1] Abele LG, Abele LG (1982). Biogeography. The biology of crustacea 1; systematics, the fossil record, and biogeography.

[ref-2] Abele LG, Kim W (1986). An illustrated guide to the marine decapod crustaceans of Florida. State of Florida, Department of Environmental Regulation, Technical Series.

[ref-3] Aguirre-Urreta MB (1990). Nuevos crustáceos decápodos (Brachyura y Anomura) en el Terciario de Patagonia, Argentina.

[ref-4] Alves DFR, Barros-Alves SDP, Teixeira GM, Cobo VJ (2012). Mithracinae (Decapoda: Brachyura) from the Brazilian coast: review of the geographical distribution and comments on the biogeography of the group. Nauplius.

[ref-5] Artal P, Van Bakel BWM, Onetti A, Fraaije RHB, Hyz˘ný M, Jagt JWM, Krobicki M, Van Bakel BWM (2014). A new inachid crab (Brachyura, Majoidea) from the Middle Eocene of the provinces of Barcelona and Girona (Catalonia, Spain). Proceedings of the 5th symposium on Mesozoic and Cenozoic decapod crustaceans, Krakow, Poland, 2013: a tribute to Pál Mihály Müller.

[ref-6] Bachmayer F (1953). Die Dekapodenfauna des tortonischen Leithakalkes von Deutsch-Altenburg (Niederösterreich). Mitteilungen der Geologischen Gesellschaft in Wien.

[ref-7] Bell T (1836). Some account of the Crustacea of the coasts of South America, with descriptions of new genera and species; founded principally on the collections obtained by Mr Cuming and Mr Miller. The Transactions of the Zoological Society of London.

[ref-8] Bertini G, Fransozo A, De Melo GA (2004). Biodiversity of brachyuran crabs (Crustacea: Decapoda) from non-consolidated sublittoral bottom on the northern coast of São Paulo State, Brazil. Biodiversity & Conservation.

[ref-9] Beschin C, Busulini A, De Angeli A, Tessier G (2007). I Decapodi dell’Eocene inferiore di Contrada Gecchelina (Vicenza-Italia settentrionale) (Anomura e Brachyura). Museo di Archeologia e Scienze Naturali “G. Zannato”, Montecchio Maggiore (Vicenza).

[ref-10] Beschin C, Busulini A, Tessier G (2015). Nuova segnalazione di crostacei associati a coralli nell’Eocene inferiore dei Lessini orientali (Vestenanova-Verona). Lavori Società Veneziana di Scienze Naturali.

[ref-11] Beschin C, De Angeli A, Checchi A (2001). Crostacei decapodi associati a coralli della “Formazione di Castelgomberto” (Oligocene) (Vicenza-Italia settentrionale). Studi e Ricerche - Associazione Amici del Museo - Museo civico “G. Zannato” - Montecchio Maggiore (Vicenza).

[ref-12] Blow WC (2003). New brachyuran crabs (Crustacea: Decapoda) from the Upper Pliocene Yorktown Formation of southeastern Virginia. Proceedings of the Biological Society of Washington.

[ref-13] Blow WC, Manning RB (1996). Preliminary description of 25 new decapod crustaceans from the middle Eocene of the Carolinas, USA. Tulane Studies in Geology and Paleontology.

[ref-14] Breton G (2009). Description of *Priscinachus elongatus* n. gen., n. sp., and Priscinachidae n. fam. for the earliest spider crab (Crustacea, Decapoda, Majoidea), from the French Cretaceous (Cenomanian). Geodiversitas.

[ref-15] Casadío S, Feldmann RM, Parras A, Schweitzer CE (2005). Miocene fossil Decapoda (Crustacea: Brachyura) from Patagonia, Argentina, and their paleoecological setting. Annals of Carnegie Museum.

[ref-16] Cobo VJ, Alves DF (2009). Relative growth and sexual maturity of the spider crab, *Mithrax tortugae* Rathbun, 1920 (Brachyura, Mithracidae) on a continental island off the southeastern Brazilian coast. Crustaceana.

[ref-17] Coelho PA, Torres MFA (1990). Revisão das espécies do gênero *Mithraculus* White na costa Atlântica da América do Sul (Crustacea, Decapoda, Majidae). Anais Sociedade Norestina de Zoologica.

[ref-18] Collins JSH, Donovan SK (2012). Pleistocene decapod crustaceans of eastern Jamaica. Caribbean Journal of Science.

[ref-19] Collins JSH, Donovan SK, Dixon HL (1996). Crabs and barnacles (Crustacea: Decapoda and Cirripedia) from the Late Pleistocene Port Morant Formation of southeast Jamaica. Bulletin of the Mizunami Fossil Museum.

[ref-20] Collins JSH, Donovan SK, Stemann TA (2009). Fossil Crustacea of the Late Pleistocene Port Morant Formation, west Port Morant Harbour, southeastern Jamaica. Scripta Geologica.

[ref-21] Collins JSH, Donovan SK, Stemann TA, Blissett DJ (2010). Crustaceans of the upper Miocene August Town Formation of southeastern Jamaica. Scripta Geologica, Special Issue.

[ref-22] Collins JSH, Fraaye RHB, Jagt JWM (1995). Late Cretaceous anomurans and brachyurans from the Maastrichtian type area. Acta Palaeontologica Polonica.

[ref-23] Collins JSH, Garvie CL, Mellish CJT (2014). Some decapods (Crustacea; Brachyura and Stomatopoda) from the Pleistocene Beaumont Formation of Galveston, Texas. Scripta Geologica.

[ref-24] Collins JSH, Morris SF (1976). Tertiary and Pleistocene crabs from Barbados and Trinidad. Palaeontology.

[ref-25] Collins JSH, Portell RW, Donovan SK (2009). Decapod crustaceans from the Neogene of the Caribbean: diversity, distribution and prospectus. Scripta Geologica.

[ref-26] De Angeli A, Beschin C (2008). Crostacei decapodi dell’Oligocene di Soghe e Valmarana (Monti Berici, Vicenza-Italia settentrionale). Studi e Ricerche - Associazione Amici del Museo - Museo Civico G. Zannato - Montecchio Maggiore (Vicenza).

[ref-27] De Grave S, Pentcheff ND, Ahyong ST, Chan T-Y, Crandall KA, Dworschak PC, Felder DL, Feldmann RM, Fransen CHJM, Goulding LYD, Lemaitre R, Low MEY, Martin JW, Ng PKL, Schweitzer CE, Tan SH, Tshudy D, Wetzer R (2009). A classification of living and fossil genera of decapods crustaceans. Raffles Bulletin of Zoology.

[ref-28] De Jesús Gómez-Cruz A, Bermúdez HD, Vega FJ (2015). A new species of *Diaulax* Bell, 1863 (Brachyura: Dialucidae) in the Early Cretaceous of the Rosablanca Formation, Colombia. Boletín de la Sociedad Geológica Mexicana.

[ref-29] De Saint Laurent M (1980). Sur la classification et la phylogénie des Crustacés Décapodes Brachyoures. I. Podotremata Guinot, 1977, et Eubrachyura sect. nov. Comptes Rendus Hebdomadaires des Séances de l’Académie des Sciences, Paris (D).

[ref-30] De Saussure MH (1853). Description de quelques crustacés nouveaux de la côte occidentale du Mexique. Revue et Magasin de Zoologie pure et appliquée (série 2).

[ref-31] De Saussure MH (1857). Diagnoses de quelques crustacés nouveaux de l’Amérique tropicale. Revue et Magasin de Zoologie pure et appliquée (série 2).

[ref-32] Desbonne I, Schramm A (1867). Brachyures. Crustacés de la Guadeloupe d’aprés un Manuscrit du Docteur Isis Desbonne compare avec les Échantillons de Crustacés de sa Collection et les Derniéres Publications de M.M. Henri de Saussure et William Stimpson. Premiere partie.

[ref-33] Desmarest AG, Cuvier F (1823). Malacostracés, *Malacostraca.*(Crust.). [Malacostracés 211–285]. Dictionnaire des Sciences Naturelles, dans lequel on Trait Méthodiquement des Différens étres de la Nature, Considérés soit en Eux-mêmes, d’après l’état Actuel de nos Connoissances, soit Relativement a l’utilité qu’en Peuvent retirer la Médecine, l’Agriculture, le Commerce et les Arts. Suivi d’une Biographie des plus Célèbres Naturalistes. Ouvrage Destiné aux Médecins, aux Agriculteurs, aux Commeŗcans, aux Artistes, aux Manufacturiers, et à tous Ceux qui ont Intérêt à Connoître les Productions de la Nature, leurs Caractères Génériques et Spécifiques, leur lieu Natal, leurs Propiétés et leurs Usages.

[ref-34] Fabricius JC (1793). Entomologiae systematica emendata et aucta, secundum Classes, Ordines, Genera, Species, adjectis Synonimis, Locis, Observationibus, Descriptionibus.

[ref-35] Felder DL, Álvarez F, Goy JW, Lemaitre R, Felder DL, Camp DK (2009). Decapoda (Crustacea) of the Gulf of Mexico, with comments on the Amphionidacea. Gulf of Mexico: origin, waters, and biota.

[ref-36] Felder DL, Thoma BP, Schmidt WE, Sauvage T, Self-Krayesky SL, Chistoserdov A, Bracken-Grissom HD, Fredericq S (2014). Seaweeds and decapod crustaceans on Gulf deep banks after the Macondo oil spill. BioScience.

[ref-37] Feldmann RM (1994). *Antarctomithrax thomsoni*, a new genus and species of crab (Brachyura; Majidae) from the La Meseta Formation (Eocene) of Seymour Island, Antarctica. Journal of Paleontology.

[ref-38] Feldmann RM, Schweitzer CE, Baltzly LM, Bennett OA, Jones AR, Mathias FF, Weaver KL, Yost SL (2013). New and previously known decapod crustaceans from the Late Cretaceous of New Jersey and Delaware, USA. Bulletin of the Mizunami Fossil Museum.

[ref-39] Feldmann RM, Schweitzer CE, Bennett O, Franţescu OD, Resar N, Trudeau A (2011). New Eocene Brachyura (Crustacea: Decapoda) from Egypt. Neues Jahrbuch für Geologie und Paläontologie Abhandlungen.

[ref-40] Fraaije RHB (2003). Evolution of reef-associated decapod crustaceans through time, with particular reference to the Maastrichtian type area. Contributions to Zoology.

[ref-41] Fraaije RHB, Menkveld-Gfeller UE, Van Bakel BWM, Jagt JWM (2010). Decapod crustaceans from the type area of the Helvetian Stage (lower Miocene) in the Bern area, Switzerland. Bulletin of the Mizunami Fossil Museum.

[ref-42] Franţescu AL (2013). Comparative study of the Eocene fossil decapod crustaceans of the North American Atlantic coast and European Tethyan provinces. D. Philosophy thesis.

[ref-43] Garth JS (1940). Some new species of brachyuran crabs from Mexico and the Central and South American mainland. Allan Hancock Pacific Expeditions.

[ref-44] Garth JS (1946). Littoral brachyuran fauna of the Galapagos Islands. Allan Hancock Pacific Expeditions.

[ref-45] Garth JS (1958). Brachyura of the Pacific coast of America: Oxyrhyncha. Allan Hancock Pacific Expeditions.

[ref-46] Gatt M, De Angeli A (2010). A new coral-associated decapod assemblage from the Upper Miocene (Messinian) Upper Coralline Limestone of Malta (Central Mediterranean). Palaeontology.

[ref-47] Gerstaecker A (1857). Carcinologishes beiträge. Archiv für Naturgeschichte.

[ref-48] Giraldes BW, Coelho Filho PA, Smyth DM (2015). Decapod assemblages in subtidal and intertidal zones—importance of scuba diving as a survey technique in tropical reefs, Brazil. Global Ecology and Conservation.

[ref-49] Glassell SA (1936). The Templeton Crocker Expedition. I. Six new brachyuran crabs from the Gulf of California. Zoologica; Scientific Contributions of the New York Zoological Society.

[ref-50] Griffin DJG (1966). A review of the Australian majid spider crabs (Crustacea, Brachyura). Australian Zoologist.

[ref-51] Guinot D (2011). The position of the Hymenosomatidae MacLeay, 1838, within the Brachyura (Crustacea, Decapoda). Zootaxa.

[ref-52] Guinot D, Tavares M, Castro P (2013). Significance of the sexual openings and supplementary structures on the phylogeny of brachyuran crabs (Crustacea, Decapoda, Brachyura), with new nomina for higher-ranked podotreme taxa. Zootaxa.

[ref-53] Herbst JFW (1782–1804). Versuch einer naturgeschichte der krabben und krebse, nebst einer systematischen beschreibung ihrer verschiedenen arten.

[ref-54] Hyžný M, Schlögl J (2011). An early Miocene deep-water decapod crustacean faunule from the Vienna Basin (Western Carpathians, Slovakia). Palaeontology.

[ref-55] Jakobsen SL, Collins JSH (1997). New middle Danian species of anomurans and brachyuran crabs from Fakse, Denmark. Bulletin of the Geological Society of Denmark.

[ref-56] Kato H, Karasawa H (1998). Pleistocene Fossil Decapod Crustacea from the Boso Peninsula, Japan. Natural History Research, Special Issue.

[ref-57] Kingsley JS (1879). On a collection of Crustacea from Virginia, North Carolina, and Florida, with a revision of the genera of Crangonidae and Palaemonidae. Proceedings of the Academy of Natural Sciences of Philadelphia.

[ref-58] Klompmaker AA (2013). Extreme diversity of decapod crustaceans from the mid-Cretaceous (late Albian) of Spain: implications for Cretaceous decapod paleoecology. Cretaceous Research.

[ref-59] Klompmaker AA, Artal P, Gulisano G (2011). The Cretaceous crab *Rathbunopon*: revision, a new species and new localities. Neues Jahrbuch für Geologie und Paläontologie Abhandlungen.

[ref-60] Klompmaker AA, Artal P, Van Bakel BWM, Fraaije RHB, Jagt JWM (2011). Etyid crabs (Crustacea, Decapoda) from mid-Cretaceous Reefal strata of Navarra, northern Spain. Palaeontology.

[ref-61] Klompmaker AA, Feldmann RM, Schweitzer CE (2012). A hotspot for Cretaceous goniodromitids (Decapoda: Brachyura) from reef associated strata in Spain. Journal of Crustacean Biology.

[ref-62] Klompmaker AA, Hyžný M, Jakobsen SL (2015). Taphonomy of decapod crustacean cuticle and its effect on the appearance as exemplified by new and known taxa from the Cretaceous–Danian crab *Caloxanthus*. Cretaceous Research.

[ref-63] Klompmaker AA, Hyžný M, Portell RW, Kowalewski M (2015). Growth, inter-and intraspecific variation, palaeobiogeography, taphonomy and systematics of the Cenozoic ghost shrimp *Glypturus*. Journal of Systematic Palaeontology.

[ref-64] Klompmaker AA, Ortiz JD, Wells NA (2013). How to explain a decapod crustacean diversity hotspot in a mid-Cretaceous coral reef. Palaeogeography, Palaeoclimatology, Palaeoecology.

[ref-65] Klompmaker AA, Schweitzer CE, Feldmann RM, Kowalewski M (2013). The influence of reefs on the rise of Mesozoic marine crustaceans. Geology.

[ref-66] Kočová Veselská M, Kočí T, Kubajko M (2014). Dynomenid crabs (Decapoda, Brachyura) and stalked barnacles (Cirripedia, Scalpelliformes) from upper Cenomanian-lower Turonian nearshore, shallow-water strata in the Bohemian Cretaceous Basin, Czech Republic. Scripta Geologica.

[ref-67] Kornecki KM (2014). Cretaceous confluence in the Coon Creek Formation of Mississippi and Tennessee, USA: taphonomy and systematic paleontology of a decapod Konsentrat-Lagerstätte. MS Thesis.

[ref-68] Krobicki M, Zatoń M (2008). Middle and Late Jurassic roots of brachyuran crabs: palaeoenvironmental distribution during their early evolution. Palaeogeography, Palaeoclimatology, Palaeoecology.

[ref-69] Lamarck JBPA (1818). Histoire naturelle des animaux sans vertèbres, présentant les caractères généraux et particuliers de ces animaux, leur distribution, leurs classes, leurs familles, leurs genres et la citation des principales espèces qui s’y rapportent; précédé d’une introduction offrant la détermination des caractères essentiels de l’animal, sa distinction du végétal et des autres corps naturels, enfin l’exposition des principes fondamentaux de la zoologie.

[ref-70] Latreille PA (1802–1803). Histoire naturelle, générale et particulière des crustacés et des insects.

[ref-71] Leach WE (1817). The zoological miscellany; being descriptions of new, or interesting animals.

[ref-72] Linnaeus C (1758). Systema naturae per regna tria naturae, secundum classes, ordines, genera, species, cum characteribus, differentiis, synonymis locis.

[ref-73] Lörenthey E, Beurlen K (1929). Die fossilen Dekapoden der Länder der Ungarischen Krone. Geologica Hungarica, Series Palaeontologica.

[ref-74] Luque J, Feldmann RM, Schweitzer CE, Jaramillo C, Cameron CB (2012). The oldest frog crabs (Decapoda: Brachyura: Raninoida) from the Aptian of northern South America. Journal of Crustacean Biology.

[ref-75] MacLeay WS, Smith A (1838). On the brachyurous decapod Crustacea brought from the Cape by Dr. Smith. Illustrations of the zoology of South Africa; consisting chiefly of figures and descriptions of the objects of natural history collected during an expedition into the interior of South Africa, in the years 1834, 1835, and 1836; fitted out by ‘The Cape of Good Hope Association for Exploring Central Africa’: together with a summary of African Zoology, and an inquiry into the geographical ranges of species in that quarter of the globe, invertebratae.

[ref-76] Mantelatto FLM, Faria FCR, Biagi R, Melo GAS (2004). Majoid crabs community (Crustacea: Decapoda) from infralittoral rocky/sandy bottom of Anchieta Island, Ubatuba, Brazil. Brazilian Archives of Biology and Technology.

[ref-77] Martins-Neto RG, Dias Júnior SC (2007). The Brazilian paleodecapod fauna: state of the knowledge. Memorie della Società Italiana di Scienze Naturali e del Museo Civico di Storia Naturale di Milano.

[ref-78] Melo GAS (1990). Descrição de *Mithrax (Mithrax) besnardi*, sp.n., Espécie abissalbe˜ntica de ãguas meridionais da América do Sul (Crustacea, Brachyura, Majidae). Atlantica.

[ref-79] Melo GAS (1996). Manual de identificação dos Brachyura (caranguejos e siris) do litoral brasileiro.

[ref-80] Melo GAS, Young PS (1998). Malacostraca—Eucarida. Brachyura. Oxyrhyncha and Brachyrhyncha. Catalogue of crustacea of Brazil.

[ref-81] Milne-Edwards H (1832). Observations sur les crustacés du genre *Mithrax*. Magasin de Zoologie.

[ref-82] Milne-Edwards A (1873–1880). Études sur les Xiphosures et les Crustacés de la région Mexicaine, mission scientifique au Mexique et dans l’Amerique centrale. Recherches zoologiques a l’histoire de la faune de l’Amerique Centrale et du Mexique, part 5.

[ref-83] Milne-Edwards A (1878). Études sur les Crustacés Podophthalmaries de la región méxicaine. Misión Scientifique du Mexique et dans l’Amerique Centrale.

[ref-84] Mitchell SF (2004). Lithostratigraphy and palaeogeography of the White Limestone Group. Cainozoic Research.

[ref-85] Morris SF (1993). The fossil arthropods of Jamaica. Geological Society of America Memoir.

[ref-86] Müller P (1974). Decapoda (Crustacea) fauna a budapesti miocénböl (2). Földtani Közlöny.

[ref-87] Müller P (1984). Decapod Crustacea of the Badenian. Geologica Hungarica, Series Palaeontologica.

[ref-88] Müller P (1996). Middle Miocene decapod Crustacea from southern Poland. Prace Muzeum Ziemi.

[ref-89] Ng PKL, Guinot D, Davie PJF (2008). Systema Brachyurorum: Part 1. An annotated checklist of extant brachyuran crabs of the world. Raffles Bulletin of Zoology.

[ref-90] Ng PKL, Jeng MS (1999). The Hymenosomatidae (Decapoda, Brachyura) of Taiwan. Crustaceana.

[ref-91] Ossó À, Díaz Isa M (2014). *Cantabroxanthus loredoensis* new genus, new species (Decapoda, Brachyura, Etyoidea) from the Middle Campanian of Loredo, Ribamontán al Mar, (Cantabria, northern Spain). Boletín de la Sociedad Geológica Mexicana.

[ref-92] Portell RW (2004). Eocene, Oligocene, and Miocene decapod crustaceans. Florida Fossil Invertebrates.

[ref-93] Portell RW, Collins JSH (2004). Decapod crustaceans of the Lower Miocene Montpelier Formation, White Limestone Group of Jamaica. Cainozoic Research.

[ref-94] Provenzano AJ, Brownell WN (1977). Larval and early post-larval stages of the West Indian spider crab, *Mithrax spinosissimus* (Lamarck) (Decapoda: Majidae). Proceedings of the Biological Society of Washington.

[ref-95] Rathbun MJ (1892). Catalogue of the crabs of the family Periceridae in the US National Museum. Proceedings of the United States National Museum.

[ref-96] Rathbun MJ (1901). The Brachyura and Macrura of Porto Rico. Bulletin of the United States Fish Commission [for 1900].

[ref-97] Rathbun MJ (1920). New species of spider crabs from the Straits of Florida and Caribbean Sea. Proceedings of the Biological Society of Washington.

[ref-98] Rathbun MJ (1925). The spider crabs of America. Bulletin of the United States National Museum.

[ref-99] Rathbun MJ (1935). Fossil Crustacea of the Atlantic and Gulf Coastal Plain. Geological Society of America, (special paper).

[ref-100] Rathbun MJ (1945). Decapod Crustacea. Geology of Lau, Fiji.

[ref-101] Roberts HB (1962). The upper Cretaceous decapod crustaceans of New Jersey and Delaware. Bulletin of the New Jersey Division of Geology.

[ref-102] Robins CM, Feldmann RM, Schweitzer CE (2013). Nine new genera and 24 new species of the Munidopsidae (Decapoda: Anomura: Galatheoidea) from the Jurassic Ernstbrunn Limestone of Austria, and notes on fossil munidopsid classification. Annalen des Naturhistorischen Museums in Wien, Serie A.

[ref-103] Samouelle G (1819). The entomologist’s useful compendium, or an introduction to the British insects, etc.

[ref-104] Schweitzer CE, Feldmann RM (2011). Revision of some fossil podotrematous Brachyura (Homolodromiidae; Longodromitidae; Torynommidae). Neues Jahrbuch für Geologie und Paläontologie Abhandlungen.

[ref-105] Schweitzer CE, Feldmann RM, Garassino A, Karasawa H, Schweigert G (2010). Systematic list of fossil decapod crustacean species. Crustaceana Monographs.

[ref-106] Starzyk N, Krzemińska E, Krzemiński W (2012). A new crab species from the Oxfordian of Poland (Decapoda: Brachyura: Goniodromitidae). Neues Jahrbuch für Geologie und Paläontologie Abhandlungen.

[ref-107] Steele DH (1988). Latitudinal variations in body size and species diversity in marine decapod crustaceans of the continental shelf. Internationale Revue der gesamten Hydrobiologie und Hydrographie.

[ref-108] Stepp AM (2014). Descriptions and biodiversity of decapods in the Seroe Domi Formation of Curaçao. M.S. thesis.

[ref-109] Stimpson W (1860). Notes on North American Crustacea, in the Museum of the Smithsonian Institution, No. 2. Annals of the Lyceum of Natural History of New York.

[ref-110] Stimpson W (1871). Preliminary report on the Crustacea dredged in the Gulf Stream in the Straits of Florida by L. F. de Pourtalés, Assist. United States Coast Survey. Part I. Brachyura. Bulletin of the Museum of Comparative Zoology at Harvard College.

[ref-111] Tavares MS, Albuquerque EF (1993). On the occurrence of *Mithrax (Mithrax) tortugae* Rathbun (Crustacea: Brachyura: Majidae) on the Brazilian continental shelf. Boletín del Instituto Oceanográfico de Venezuela.

[ref-112] Tavares M, Santana W (2015). A new genus and two new species of hymenosomatid crabs (Crustacea: Brachyura: Hymenosomatidae) from the southwestern Atlantic and eastern Australia. Zootaxa.

[ref-113] Távora VA, Paixão GMC, Da Silva FA (2010). Considerações filogenéticas e biogeografia histórica dos malacostráceos (decápodes e isópodes) cenozóicos do Brasil. Revista Brasileira de Geociências.

[ref-114] Todd JA, Collins JSH (2005). Neogene and Quaternary crabs (Crustacea, Decapoda) collected from Costa Rica and Panama by members of the Panama Paleontology Project. Bulletin of the Mizunami Fossil Museum.

[ref-115] Türkay M (1986). *Mithrax caboverdianus* n. sp., eine neue Seespinnen-Art von den Kapverdischen Inseln (Crustacea: Decapoda: Brachyura: Majidae). Courier Forschungsinstitut Senckenberg.

[ref-116] Varela C (2013). Nuevos datos sobre los crustáceos fósiles (Decapoda: Brachyura) de Cuba. Solenodon.

[ref-117] Varela C, Rojas-Consuegra R (2009). Crustáceos (Decapoda: Brachyura) fósiles de Cuba. Solenodon.

[ref-118] Varela C, Rojas-Consuegra R (2011). El registro fósil de los crustáceos decápodos (Arthropoda, Crustacea) marinos de Cuba.

[ref-119] Vega FJ, Feldmann RM, Sour-Tovar F (1995). Fossil crabs (Crustacea: Decapoda) from the Late Cretaceous Cárdenas Formation, east-central Mexico. Journal of Paleontology.

[ref-120] Vega FJ, Gholamalian H, Hassani MJ, Sajadi SH, Schaaf P (2012). Miocene Crustacea from northern Bandar Abbas, South Iran. Neues Jahrbuch für Geologie und Paläontologie Abhandlungen.

[ref-121] Verrill AE (1908). Decapod Crustacea of Bermuda; I. Brachyura and Anomura. Their distribution, variations and habits. Transactions of the Connecticut Academy of Arts and Sciences.

[ref-122] Wagner HP (1990). The genera *Mithrax* Latreille, 1818 and *Mithraculus* White, 1847 (Crustacea: Brachyura: Majidae) in the Western Atlantic Ocean. Zoologische Verhandelingen.

[ref-123] Wicksten MK (1993). A review and a model of decorating behavior in spider crabs (Decapoda, Brachyura, Majidae). Crustaceana.

[ref-124] Williams AB (1984). Shrimps, lobsters, and crabs of the Atlantic coast of the eastern United States, Maine to Florida.

[ref-125] Windsor AM (2010). Phylogeny of the superfamily Majoidea (Crustacea: Decapoda: Brachyura) with an emphasis on the family Inachoididae and subfamily Mithracinae. D. Phil. thesis.

[ref-126] Windsor AM, Felder DL (2009). Re-evaluation of species allied to *Mithrax hispidus* (Decapoda: Brachyura: Majoidea: Mithracidae) based on three mitochondrial genes. Zootaxa.

[ref-127] Windsor AM, Felder DL (2014). Molecular phylogenetics and taxonomic reanalysis of the family Mithracidae Macleay (Decapoda : Brachyua : Majoidea). Invertebrate Systematics.

[ref-128] Young P, Chandler R (2014). Neogene and Quaternary decapods. Fossil invertebrates and plants—volume I of IV.

